# Bacterial Metabolism-Initiated Nanocatalytic Tumor Immunotherapy

**DOI:** 10.1007/s40820-022-00951-0

**Published:** 2022-11-11

**Authors:** Wencheng Wu, Yinying Pu, Shuang Gao, Yucui Shen, Min Zhou, Heliang Yao, Jianlin Shi

**Affiliations:** 1 The State Key Lab of High Performance Ceramics and Superfine Microstructures, Shanghai Institute of Ceramics, Chinese Academy of Sciences; Research Unit of Shanghai Nanocatalytic Medicine in Specific Therapy for Serious Disease, Chinese Academy of Medical Sciences (2021RU012), Shanghai, 200050 People’s Republic of China; 2 Department of Medical Ultrasound, Shanghai Tenth People’s Hospital, Ultrasound Research and Education Institute, Tongji University Cancer Center, Tongji University School of Medicine, Shanghai, 200072 People’s Republic of China; 3 Digestive Endoscopy Center, Shanghai Fourth People’s Hospital to Tongji University, Shanghai, 200081 People’s Republic of China

**Keywords:** Bacterial metabolism, In situ nanocatalytic therapy, Immunotherapy

## Abstract

The low immunogenicity of tumors remains one of the major limitations of cancer immunotherapy. Herein, we report a bacterial metabolism-initiated and photothermal-enhanced nanocatalytic therapy strategy to completely eradicate primary tumor by triggering highly effective antitumor immune responses. Briefly, a microbiotic nanomedicine, designated as Cu_2_O@ΔSt, has been constructed by conjugating PEGylated Cu_2_O nanoparticles on the surface of an engineered Salmonella typhimurium strain (ΔSt). Owing to the natural hypoxia tropism of ΔSt, Cu_2_O@ΔSt could selectively colonize hypoxic solid tumors, thus minimizing the adverse effects of the bacteria on normal tissues. Upon bacterial metabolism within the tumor, Cu_2_O@ΔSt generates H_2_S gas and other acidic substances in the tumor microenvironment (TME), which will in situ trigger the sulfidation of Cu_2_O to form CuS facilitating tumor-specific photothermal therapy (PTT) under local NIR laser irradiation on the one hand. Meanwhile, the dissolved Cu^+^ ions from Cu_2_O into the acidified TME enables the nanocatalytic tumor therapy by catalyzing the Fenton-like reaction of decomposing endogenous H_2_O_2_ into cytotoxic hydroxyl radicals (·OH) on the other hand. Such a bacterial metabolism-triggered PTT-enhanced nanocatalytic treatment could effectively destroy tumor cells and induce a massive release of tumor antigens and damage-associated molecular patterns, thereby sensitizing tumors to checkpoint blockade (ICB) therapy. The combined nanocatalytic and ICB therapy results in the much-inhibited growth of distant and metastatic tumors, and more importantly, induces a powerful immunological memory effect after the primary tumor ablation.

**Supplementary Information:**

The online version contains supplementary material available at 10.1007/s40820-022-00951-0.

## Highlights

- Novel-designed microbiotic nanomedicine capable of targeting hypoxic tumor sites.
- First paradigm of bacterial metabolism-initiated and photothermal therapy-enhanced nanocatalytic therapy for ablating tumors and inducing immunogenic cell death.
- Strong antitumor immunity activation for abscopal tumors therapy.

**Supplementary Information:**

The online version contains supplementary material available at 10.1007/s40820-022-00951-0.

## Graphical abstract

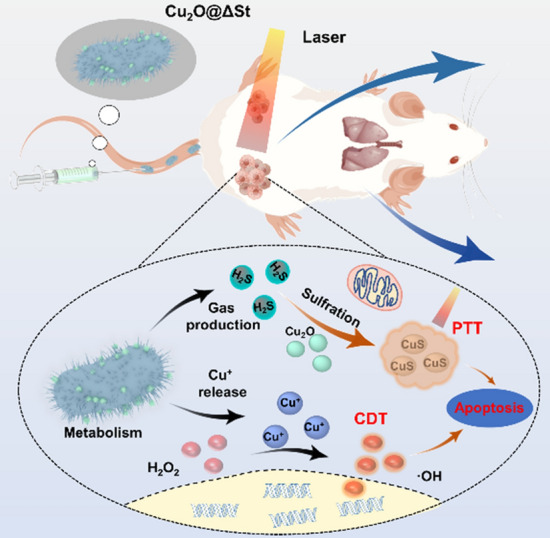

## Introduction

The advances in cancer immunotherapy have brought new opportunities to the field of oncology [1–3]. Although therapies that block T-cell immunosuppressive pathways, such as PD-1/PD-L1 and CTLA-4 inhibitors, can activate the adaptive immune system and have achieved unprecedented clinical advances, overall response rates in the treated patients remain low at the current stage [4–6]. Crafty malignant cells can easily evade the surveillance, recognition, and attack of the immune system by leveraging various strategies, resulting in the failure of antitumor immunotherapies [7–9]. To enhance the immunogenicity of solid tumors and remodel the immunosuppressive tumor microenvironment (ITME), a novel while highly attractive option is to activate immunogenic cell death (ICD) by utilizing dying cancer cells as therapeutic vaccines for antitumor immune response stimulation [10–12]. Various therapeutic approaches, such as thermal ablation therapy and reactive oxygen species (ROS)-based nanocatalytic tumor therapy, have been employed to induce ICD [13–15]. Plentiful damage-associated molecular patterns (DAMPs) including high mobility group box 1 (HMGB1), heat shock protein 70 (HSP70) as well as calreticulin (CRT) could be released or expressed upon ICD [16]. Based on strengthened antigen presentation of dendritic cells (DCs) and the activation of cytotoxic T lymphocytes (CD8^+^ T cells), these danger signals further boost the autologous immune system to fight cancer [17].

Photothermal therapy (PTT) has gained widespread interest as an emerging strategy to directly ablate tumors and further elicit ICD in cancer cells under local near-infrared (NIR) light irradiation [18–20]. However, some unexpected side effects of PTT, such as inflammation caused by the indiscriminate thermal effect of conventional “always-on” photothermal agents, may contribute to the recurrence and metastasis of cancer. Harnessing unique biomarkers within TME, multiple stimuli-activatable photothermal agents have been developed for the accurate and efficient tumor-specific PTT [21–23]. In particular, recent studies have reported that cuprous oxide (Cu_2_O) nanoparticles can be sulfidated by endogenous hydrogen sulfide (H_2_S) that is highly expressed in colon cancer, to in situ form CuS possessing photoacoustic imaging (PAI) and photothermal conversion properties [24]. However, the amount of endogenous H_2_S is rather limited and only overexpressed in colon cancer, while exogenous H_2_S donors always suffer from chemical instability and poor tumor penetration, which greatly diminishes the effectiveness and universality of this in situ PTT strategy [25–27]. Interestingly, attenuated Salmonella, a facultative anaerobic Gram-negative bacteria, not only possesses the exceptional tumor colonizing ability but also exhibits distinctive metabolic activities that allow the generation of a substantial amount of H_2_S and acidic substance via anaerobic respiration using sulfur compounds as terminal electron acceptors [28–30]. Such inherently tantalizing properties also endow Salmonella with unprecedented potential for antitumor immunotherapy based on its unique metabolism features.

**Scheme 1:**
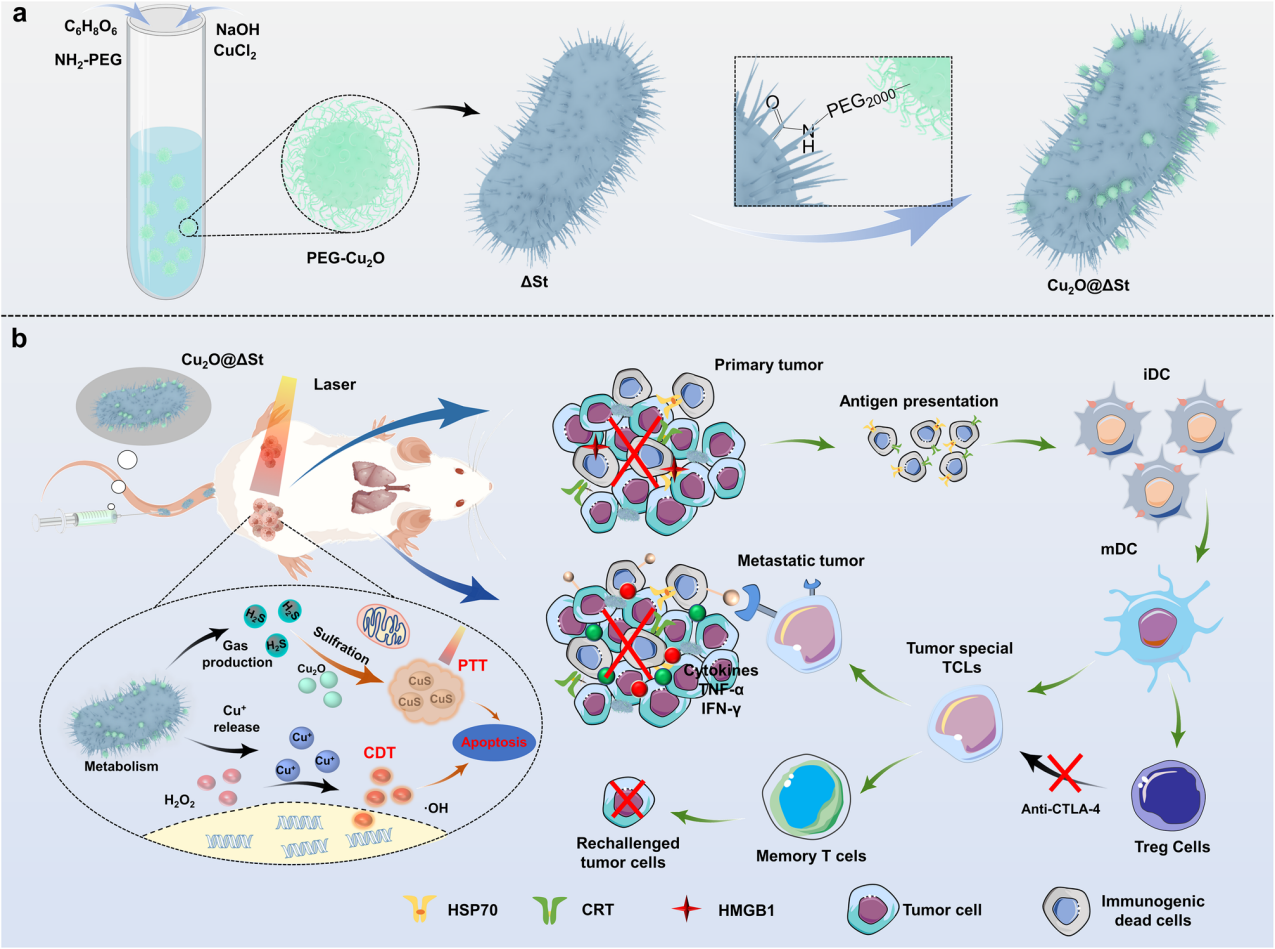
**a** Schematic diagram of the construction of Cu_2_O@ΔSt microbiotic nanomedicine by bonding Cu_2_O onto the surface of ΔSt. **b** Schematic representation of photothermal-enhanced cancer nanocatalytic therapy initiated by bacterial metabolism for triggering ICD and adaptive immune activation

In this work, we have rationally designed and prepared a novel microbiotic nanomedicine Cu_2_O@ Salmonella typhimurium strain (ΔSt) and proposed a strategy of bacterial metabolism-enabled and photothermal-enhanced nanocatalytic immunotherapy for malignant tumors. A bio-orthogonal reaction was applied to conjugate PEGylated Cu_2_O nanoparticles onto genetically engineered Salmonella typhimurium strain (ΔSt) featuring the obligate anaerobic character (**Scheme ****1****a**). Due to the innate hypoxia tropism of ΔSt, this microbiotic nanomedicine could selectively colonize tumor tissues, thus minimizing adverse effects on normal tissues. Accompanying the metabolism process, H_2_S and other acidic substances will then be produced by Cu_2_O@ΔSt, where the H_2_S can react in situ with Cu_2_O to form CuS, a typical photothermal agent, further achieving tumor-specific PTT under local NIR laser irradiation. Meanwhile, the exacerbation of the acidic TME drives the release of Cu^+^ from Cu_2_O, which offers a specific nanocatalytic therapeutic effect upon reacting with endogenous hydrogen peroxide (H_2_O_2_) to generate cytotoxic hydroxyl radicals (·OH). Bacterial metabolism-initiated and PTT-enhanced nanocatalytic treatment will destroy tumor cells and induce a massive release of tumor antigens and DAPM_S_, thereby intensifying tumor immunogenicity. When combined with immune checkpoint blockade (ICB) treatment, the growth of metastatic tumors can be significantly inhibited, and more importantly, a powerful immunological memory effect that prevents cancer recurrence can ultimately be achieved after the primary tumor ablation (**Scheme ****1****b**).

## Materials and Methods

### Materials

1-Ethyl-3(3-dimethylaminopropyl) carbodiimide (EDC), N-hydroxysuccinimide (NHS), sodium hydroxide (NaOH), ascorbic acid (AA), and copper acetate monohydrate (Cu(Ac)_2_·H_2_O) were obtained from Adamas-Beta Co. (Shanghai, China). [Amino(polyethylene glycol) 2000] (PEG-NH_2_) was obtained from Xi’an Ruixi Biological Technology Co. (Xian, China). Phosphate buffer solution (PBS), Dulbecco’s modified eagle medium (DMEM), calcein, 4, 6-diamidino-2-phenylindole (DAPI), propidium iodide (PI), fluorescein isothiocyanate (FITC), and cell counting Kit-8 (CCK-8) were purchased from United Bioresearch, Inc Co. The standard attenuated Salmonella Typhimurium (VNP20009) was bought from the American Type Culture Collection (ATCC). All of the antibodies mentioned in this article were ordered from BioLegend, Inc.

### Fabrication of PEG-Cu_2_O Nanoparticles

PEG-Cu_2_O was synthesized according to a modified method [1]. Briefly, PEG-NH_2_ (500 mg) was first dissolved in 10 mL of Cu(Ac)_2_·H_2_O (119 mg) aqueous solution. After PEG-NH_2_ was completely dissolved, 0.5 mL of NaOH solution (240 mg mL^−1^) was added to the above solution and was then further reacted for 0.5 h. Afterward, 2 mL of AA solution (0.1 M) was added and stirred for 0.5 h. Finally, the PEG-Cu_2_O nanoparticles were obtained by centrifugation. The obtained product was poured by repeatedly washing with deionized water and ethanol for further use.

### Construction of Oxygen-Sensitive Salmonella Typhimurium Mutant

Typically, an essential gene (asd) was firstly engineered so that it is controlled by a hypoxia-conditioned promoter. In this case, the normal functions of bacteria will not be disturbed by the deletion or mutation of this gene. In normal tissues under aerobic conditions, the asd gene is not expressed, so the mutant bacteria cannot synthesize diaminopimelic acid (DAP), which is essential for survival unless DAP is provided by the environment. As a result, ΔSt cannot proliferate in normal oxygenated tissues. Both the genetic modification and the screening process of mutant bacteria were completed by Haixing Biosciences company.

### Linkage of PEG-Cu_2_O and ΔSt (Cu_2_O@ΔSt)

PEG-Cu_2_O nanoparticles were linked on the surface of ΔSt via the amide condensation between the -NH_2_ in PEG-Cu_2_O and -COOH on the surface of ΔSt. Briefly, ΔSt (1 × 10^5^ CFU) was dispersed in PBS solution (1 mL), and 1 mL of PEG-Cu_2_O aqueous solution with different concentration (65, 125, and 250 μg mL^−1^) was added into bacteria suspension with 1.15 mg EDC and 1.3 mg NHS. After stirring for 0.5 h, the bacteria linked with PEG-Cu_2_O nanoparticles were obtained by centrifugation (3000 rpm, 5 min) and washed with PBS three times.

### Characterization

The hydrodynamic diameter of PEG-Cu_2_O nanoparticles in deionized water at pH 7.4 was measured by the Malvern Zetasizer Nano series (Nano ZS90). The morphology of PEG-Cu_2_O, ΔSt, and Cu_2_O@ΔSt was observed under the transmission electron microscope (TEM, JEM-2100F). The chemical component of Cu_2_O@ΔSt was obtained by energy-dispersive X-ray spectroscopy (EDS) elemental analysis. X-ray diffraction measurements (XRD Bruker D8 Focus, Bruker, Billerica, MA, USA; 2*θ* ranging from 10 to 90° Cu Kα1) were performed on as-synthesized Cu_2_O@ΔSt powders. The valence of copper in PEG-Cu_2_O nanoparticles was analyzed by X-ray photoelectron spectroscopy (XPS) spectrum (Thermo Fisher Scientific, Waltham, MA, USA).

### Detection of pH Value

At different time points (0, 0.5, 1, 2, 4, 8, 12, and 24 h), the pH values of the LB medium containing Cu_2_O@ΔSt bacteria were measured by a pH meter (INESA Scientific Instrument Co. PHSJ-6L). The free LB medium was used as a control.

### Detection of Copper Ions Release

At different time points (0, 0.5, 1, 2, 4, 8, 12, and 24 h), 1.5 mL of testing solutions were sucked out from PEG-Cu_2_O PBS solutions (30 mL,1 mg mL^−1^) with different pHs (pH = 7.4, 6.0) or LB medium containing Cu_2_O@ΔSt (30 mL,10^5^ CFU), respectively. The Cu ions content in the solution was detected by coupled plasma optical emission spectrometer (ICP-OES, Agilent Technologies, USA).

### Detection of ·OH In Vitro

TMB assay was applied to monitor the chromogenic reaction of the Cu_2_O/H_2_O_2_ system. PBS (800 μL) with different pH values (7.4, 6.0), Cu_2_O suspensions (0, 25, 50, 100, and 200 μg mL^−1^) were mixed with TMB and H_2_O_2_ at the final concentrations of 800 μM and 20 mM, respectively. Conversely, in PBS (800 μL) with different pH values (7.4, 5.0), H_2_O_2_ suspensions (0, 1.25, 2.5, 5, 10, and 20 mM) were mixed with TMB and Cu-LDH at the final concentrations of 800 μM and 200 μg mL^−1^, respectively. The absorbance (*λ* = 650 nm) of the TMB was detected by a UV–Vis spectrometer. The ESR spectrometer was used to quantitatively analyze the production of ·OH in different systems. In this assay, four groups were setted as follows: H_2_O_2_ (100 μM) only, Cu_2_O (pH = 7.4) + H_2_O_2_, Cu_2_O (pH = 6.0) + H_2_O_2_, and Cu_2_O@ΔSt + H_2_O_2_, all group shared the equivalent concentration of Cu_2_O of 20 μg mL^−1^ (200 μL). Immediately after the addition of DMPO (5 μL), the mixture was detected by an ESR spectrometer (JEOL-FA 200, Japan).

### In Vitro Cytotoxicity

4T1 cells were treated as follows: incubation with culture medium containing different concentrations of Cu_2_O for 24 h at the existence of H_2_O_2_ (100 μM); incubation with culture medium containing different concentrations of Cu_2_O at the existence of NaHS (2 μM) for 8 h, then irradiated with 808 nm laser (1.5 W cm^−2^) for 10 min and incubation for further 16 h; incubation with culture medium containing different concentrations of Cu_2_O at both the existence of NaHS (2 μM) and H_2_O_2_ (100 μM) for 8 h, then irradiated with 808 nm laser (1.5 W cm^−2^) for 10 min and incubation for further 16 h. Above, the described treatments share the same Cu_2_O concentration gradient (0, 1.25, 2.5, 5, 10, and 20 μg mL^−1^). Then, the cells in each well were washed gently with PBS solution and then incubated with CCK-8 solution (100 μL, 10% CCK-8) for another 2 h. The absorbance of each well at 450 nm was detected by the microplate reader (Bio-TekELx800). The expression of caspase 3 associated with apoptosis in tumor cells was detected by Caspase-3 Assay Kit and western blotting.

### Live/Dead Cell Staining and Immunofluorescence

4T1 cells in the culture dish were treated with the same above-mentioned treatments at the concentration of Cu_2_O of 20 μg mL^−1^. The cells treated with free DMEM were set as a control. After that, calcein acetoxymethyl ester (Calcein-AM)/propidium iodide (PI) (50 μL, 20 mM) were added and incubation for another 30 min. Finally, the live/dead cells were observed by CLSM. As for immunofluorescence, after undergoing different treatments, the cells were washed with PBS and fixed with 4% paraformaldehyde for 10 min. Immediately afterward, anti-CRT, anti -HMGB1, and anti-HSP70 were added to the cells and incubation for 2 h at room temperature, respectively. Then, the FITC or AP-conjugated secondary antibodies were added and incubation for 1 h. Finally, the cell nuclei were stained with DAPI before observing on CLSM.

### In Vitro DC Cells Stimulation Assay

The bone-marrow-derived DCs were seeded in the bottom of the transwell system, while the 4T1 cancer cells pretreated with different conditions (Control, Laser, ΔSt, Cu_2_O with H_2_O_2_, Cu_2_O with NaHS + Laser, and Cu_2_O with both H_2_O_2_ and NaHS + Laser) were put in the upper compartment to stimulate DCs for 48 h. Then, the bottom DCs were harvested and stained with anti-CD11c FITC, anti-CD80 APC, and anti-CD86 PE followed by flow cytometry FCM (BD LSRFortessa) analysis. The proinflammatory cytokines (i.e., IL-6, IL12, and TNF-α) in suspension were detected by ELISA kits with a standard protocol.

### Tumor Models

BALB/c mice (female, 4 weeks) were obtained from Beijing Vital River Laboratory Animal Technology Co. All animal experiment protocols were approved by the Laboratory Animal Center of Shanghai Tenth Peoples’ Hospital and complied with the policies of the National Ministry of Health. To generate the different tumor-bearing mouse models, PANC-1 (1 × 10^6^), CT26 (1 × 10^6^), and 4T1 (1 × 10^6^) cells were subcutaneously implanted into normal BALB/c mice.

### ΔSt Colonization In Vivo

After injection of Cu_2_O@ΔSt (10^5^ CFU), the major organs (heart, liver, spleen, lung, kidney, and tumor) of healthy and tumor-bearing mice were collected at different time points. Then, these major organs were extracted, weighed, and homogenized at 4 °C in sterile PBS (pH = 7.4). Those samples were diluted (10-, 100-, or 1000- fold) and plated on chromogenic LB plates, where ΔSt will be displayed in purple. After 24 h of incubation, bacterial colonies were counted. The bacterial titer (CFU per gram of tissue) was presented by the ratio of colony counts and tissue weights.

### In Vivo Fluorescence Imaging

When the tumor volume reached about 200 mm^3^, the tumor-bearing mice were injected intravenously with IR783 labeled Cu_2_O@ΔSt (10^5^ CFU). Then, the IR783 fluorescence signals over time were detected by the ex/in vivo imaging system (VISQUE Invivo Smart-LF, Korea). The major organs (heart, liver, spleen, lung, kidneys) and tumors were also collected for semiquantitative biodistribution analysis and imaging after 12-h injection.

### In Vivo Toxicity

First, a biosafety assessment experiment of Cu_2_O@ΔSt was performed by injecting different amounts of ΔSt (10^3^ 10^4^, 10^5^, and 10^6^, *n* = 5) into healthy BABL/C mice for optimizing the dose of bacteria for subsequent in vivo application. To monitor the bacterial toxicity at different stages, mice injected with ΔSt at the dose of 10^5^ CFU were sacrificed at 1, 7, and 30 days, respectively. The mice in the control group were treated with PBS. The serum of mice was collected for blood biochemistry and hemolysis analysis, and their main organs (heart, liver, lung, spleen, and kidney) were collected for hematoxylin and eosin (H&E) staining.

### In Vivo Anticancer Effect and Immune Activation Exploration

4T1, PANC-1, and CT26 tumor-bearing mice were randomly divided into six groups (n = 10), including: (1) Control, (2) Laser (808 nm, 1.5 W cm^−2^), (3) Bacterial (ΔSt, 10^5^ CFU), (4) Cu_2_O@ΔSt (10^5^ CFU), (5) Cu_2_O + Laser (20 mg kg^−1^), and (6) Cu_2_O@ΔSt + Laser (10^5^ CFU). When the tumors reached ~ 50 mm^3^, PBS was intravenously injected into the control group. On third day after treatment, tumors of mice (*n* = 5) were collected, dissected, and subjected to CRT, HSP-70, and HMGB-1 immunofluorescence staining. For immune activation exploration, the tumor-draining lymph nodes of treated mice were excised and homogenized into single-cell suspensions. Then, the anti-CD11c FITC, anti-CD80 APC, and anti-CD86 PE were used to mark the mature DCs. In addition, to analyze the activation of cytotoxicity T lymphocyte (CTL), cell suspensions were co-stained with anti-CD3-FITC, anti-CD4-PE, and anti-CD8-APC antibodies guided by the manual manufacturer’s protocols. The proinflammatory cytokines (IL-6, TNF-α, and IFN-γ) content in serum were tested by ELISA. Next, the weight and tumor volume of the remaining mice in each group were continued to be recorded (*n* = 5). In addition, tumors from mice were collected, dissected, and subjected to Ki-67 and TUNEL staining.

### Distant Tumor Inhibition

Mice were randomly divided into five groups including: (1) Control, (2) Bacterial (ΔSt), (3) Cu_2_O@ΔSt, (4) Cu_2_O + Laser (10 mg kg^−1^), and (5) Cu_2_O@ΔSt + Laser. The bacterial injection dose was at the ΔSt dose of 10^5^ CFU, while the mice of the control group were injected with PBS. For the first tumor plantation, CT26 cells (1 × 10^6^) were subcutaneously injected into the left leg of each mouse. Four days later, CT26 cells (5 × 10^5^) were subcutaneously injected into the right leg of each mouse to establish the second tumor. The first tumor was treated on day 0 as the above-described plan, and the anti-CTLA-4 (10 μg) was injected into mice in all groups on the second, fifth, and eighth day. During the treatment, the volumes of distant tumors were monitored.

### Immunoassay in Distant Tumors

To analyze the immune cells in distant tumors, those tumors were collected from mice in different groups. These obtained tumors were further homogenized into single-cell suspensions, and then, the anti-CD3-FITC, anti-CD4-PE, and anti-CD8-APC antibodies were added to label cell membrane protein phenotypes for further analysis. CTLs were CD3^+^CD4^−^CD^8+^. To analyze CD4^+^ helper T cells, anti-Foxp3-PE and anti-CD4-PerCP were further used to label those cells. According to the different phenotypes, CD4^+^ helper T cells were classified into Tregs (CD4^+^Foxp3^+^) and effector T cells (CD4^+^Foxp3^−^). Besides, TNF-α, IL6, IL12, and IFN-γ in the serum in each group were also analyzed by using ELISA kits.

### Immune Memory Evaluation

After the elimination of primary CT26 tumors by surgery or combination therapy for 40 days, CT26 cells (1 × 10^6^) were subcutaneously inoculated into those mice again and followed by intravenous injection of anti-CTLA-4 (20 μg mice^−1^) on days 43, 46, and 49. The tumor volumes were then closely recorded. Flow cytometry was used to analyze the percent of memory T cells in spleens. The harvested spleens were homogenized into single-cell suspensions and stained with anti-CD3-FITC, anti-CD8-PerCP-Cy5.5, anti-CD62L-APC, and anti-CD44-PE antibodies. Immune T cells could be classified into TCM (CD3^+^CD8^+^CD62L^+^CD44^+^) and TEM (CD3^+^CD8^+^ CD62L^−^CD44^+^) cells.

### Statistical Analysis

The in vitro tests were performed three times independently. All in vivo experiments were performed after randomization and performed with five mice per group. Statistical analyses were conducted using OriginPro 2018 software. Animal survival was calculated by the Kaplan–Meier method, and the *P* value was obtained by the log-rank test. The significance of the data is determined by the student’s test: *p < 0.05, **p < 0.01, and ***p < 0.001.

## Results and Discussion

### Fabrication of Microbiotic Nanomedicine (Cu_2_O@ΔSt)

**Fig. 1:**
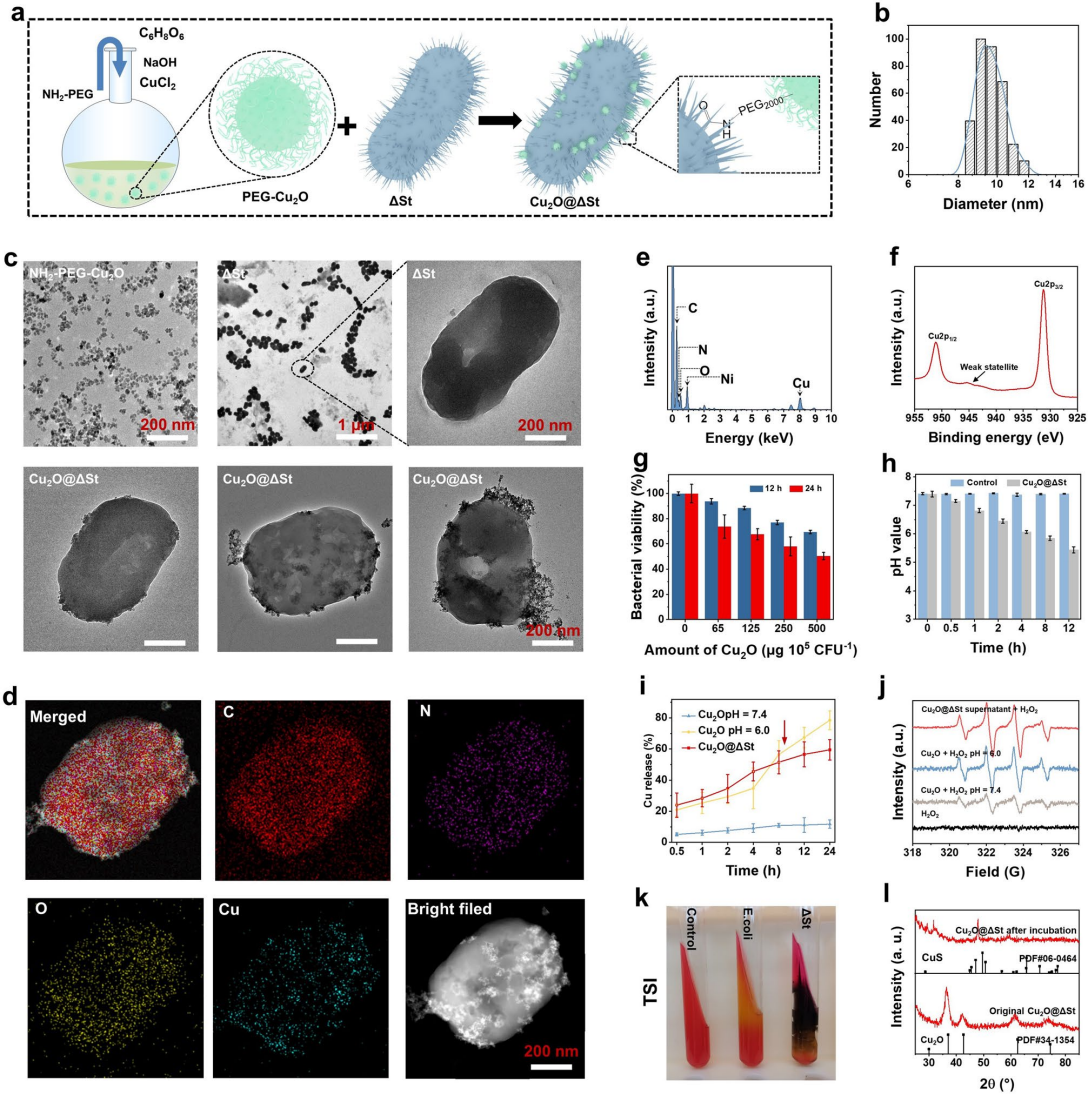
Fabrication and characterization of Cu_2_O@ΔSt. **a** Schematic illustration of the synthesis of Cu_2_O and the linkage of Cu_2_O onto ΔSt. **b** The hydrodynamic diameter of Cu_2_O determined by dynamic light scattering (DLS) detection. **c** TEM images of Cu_2_O, ΔSt, and Cu_2_O@ΔSt at the feed ratios of 65, 125, and 250 μg Cu_2_O per 10^5^ CFU ΔSt. **d** Elemental mappings of Cu_2_O@ΔSt. **e** EDS of Cu_2_O@ΔSt. **f** XPS spectra of Cu_2_O. **g** Bacterial viabilities of ΔSt treated with different feed doses of Cu_2_O for 12 and 24 h (*n* = 6, mean ± standard error). **h** The pH values at different time points of the medium with or without the addition of Cu_2_O@ΔSt (*n* = 3, mean ± standard error). **i** Accumulated Cu release from Cu_2_O@ΔSt after incubation for varied time durations (*n* = 3, mean ± standard error). **j** ESR spectra of different reaction systems. **k** Color changes of triple sugar iron agar media after planting with different bacteria. **l** XRD patterns of original Cu_2_O@ΔSt powder and that collected from Cu_2_O@ΔSt suspension after incubation for 24 h

Initially, the obligate anaerobic Salmonella typhimurium strain (ΔSt) was constructed by placing an essential gene of the standard attenuated facultative anaerobic Salmonella typhimurium strain YS1646, which controls the synthesis of necessary diaminopimelic acid (DAP) for bacterial proliferation, under the hypoxia-initiated promoter (Figs. S1a and S2) [31]. In simulated normal aerobic conditions, ΔSt synthesizes no DAP due to the silence of the asd gene and would be lysed during growth unless DAP was additionally provided, which can minimize its adverse effects on normal organs (Fig. S1b, c). In contrast, under hypoxic conditions simulating tumor tissue, the bacteria can proliferate normally with or without the additional addition of DAP (Fig. S1d, e). Then, the synthesized PEGylated Cu_2_O nanoparticles (PEG-Cu_2_O NPs) were bonded on the surface of ΔSt through the amide condensation reaction, giving a Cu_2_O modified ΔSt complex named as Cu_2_O@ΔSt, as schematically shown in Fig. 1a. The engineered bacteria exhibit a rod-like morphology of about 550 and 300 nm in length and width, respectively, providing sufficient locations for the attachment of small PEG-Cu_2_O NPs (~ 10 nm, Fig. 1b and c). The surface of the bacteria is usually enriched with N-acetylmuramic acid and N-acetylglucosamine; therefore, PEG-Cu_2_O NPs with amino groups can be readily bonded on the surface of bacteria by amide condensation reaction under the catalysis by EDC and NHS to form a novel microbiotic nanomedicine Cu_2_O@ΔSt. As TEM images display (Fig. 1c bottom), different amounts of PEG-Cu_2_O NPs could be anchored on the surface of bacteria by tuning the feed ratio between PEG-Cu_2_O NPs and ΔSt. In addition, energy-dispersive X-ray spectroscopy (EDS) and TEM elemental mapping images verify the presence of Cu, O, C, and N signals derived from Cu_2_O@ΔSt, demonstrating that Cu_2_O NPs have been successfully and uniformly anchored on the surface of bacteria (Fig. 1d, e). The combined efficiency of Cu_2_O to bacteria was calculated to be 42.5%. The high-resolution XPS spectrum of Cu 2*p* shows that the Cu species that originated from Cu_2_O are Cu^+^(Fig. 1f), which can be utilized to catalyze a Fenton-like reaction for producing high-cytotoxic ·OH [32]. The powerful peroxidase-mimicking capability of the prepared Cu_2_O in producing ·OH by decomposing H_2_O_2_ under acidic conditions has been well-demonstrated in a typical 3,3′,5,5′-tetramethylbenzidine (TMB) colorimetric assay (Fig. S3). Considering the bacterial toxicity of PEG-Cu_2_O NPs, the feed ratio between PEG-Cu_2_O and bacteria was further optimized by measuring the effect of PEG-Cu_2_O on bacterial viability. The bacterial Cell Counting Kit-8 (CCK-8) and bacterial cloning experiment results show that the excessive PEG-Cu_2_O has a significant impact on the survival of ΔSt (Figs. 1g and S4). Particularly, at a feed ratio of 500 μg 10^6^ CFU^−1^, the survival rate of the ΔSt after 24 h of incubation is rather low at 50.3%. Therefore, to maintain the high-enough bacterial alive activity, all of the Cu_2_O@ΔSt microbiotic nanomedicine used in subsequent studies were prepared at the ratio of 125 μg Cu_2_O/10^5^ CFU ΔSt.

### In Vitro Bacterial Metabolism-Initiated ·OH Generation and Sulfuration of Cu_2_O

Next, we investigated the performance of Cu_2_O@ΔSt microbiotic nanomedicine in producing ·OH and in situ sulfidate Cu_2_O in vitro. On the one hand, ΔSt is able to break down various sugar molecules to produce pyruvic acid, which can be further metabolized into various acids, eventually leading to a decreased pH value of the culture environment (Fig. 1h). The resulting acidic environment makes Cu^+^ ions be released from Cu_2_O bound on the bacterial surface, thereby catalyzing the decomposition of H_2_O_2_ to produce cytotoxic ·OH. As shown in Fig. 1i, the Cu^+^ release rate from Cu_2_O at pH 6.0 or from Cu_2_O@ΔSt is significantly higher than that from Cu_2_O at pH 7.4. Interestingly, a significant decrease in the rate of Cu release from Cu_2_O@ΔSt occurs compared to Cu_2_O under acidic conditions in 8 h of incubation, which may be due to the sulfidation of partial Cu_2_O on the bacterial surface by H_2_S generated during bacterial metabolism. The electron spin resonance (ESR) spectroscopy was applied to directly detect the production of ·OH in different reaction systems, where 5,5-dimethyl-1-pyrroline N-oxide was used as a spin trap of ·OH. The intensity of the characteristic ·OH signal detected after the reaction of Cu_2_O with H_2_O_2_ under acidic conditions is significantly stronger than that detected under the neutral condition, and the characteristic ·OH signal in the group containing only H_2_O_2_ is non-detectable (Fig. 1j). Notably, a strong ·OH signal was also detected when the substrate H_2_O_2_ was added to the Cu_2_O@ΔSt supernatant after 8 h of incubation, indicating that bacterial metabolism has initiated the release of Cu^+^ for triggering ·OH generation. On the other hand, ΔSt is naturally able to produce H_2_S by decomposing sulfur amino acids, which sulfide Cu_2_O into CuS for cancer photothermal ablation. To prove this, firstly, the typical trisaccharide iron test assay was performed to demonstrate the capacity of ΔSt in producing H_2_S. Triple sugar iron agar is often used to investigate the sugar decomposition for H_2_S production by the bacteria, which can be presented by its color change after incubation with bacteria. As Fig. 1k presents, the agar inoculated with ΔSt shows a staggered yellow and black color compared to the bacteria-free agar, whereas E. coli only gives the agar a yellow color. This color difference demonstrates that ΔSt is capable of producing H_2_S in addition to other acidic substances during metabolism, as further evidenced by the H_2_S detection kit assay (Fig. S5). Moreover, in the XRD pattern (Fig. 1l), the peaks of the Cu_2_O@ΔSt powder well-match those of cubic Cu_2_O. However, in 24 h of incubation, a weak XRD peak belonging to CuS can be found in the collected powder residue, proving that the Cu_2_O NPs bound on the bacterial surface have been sulfidated by the generated H_2_S. Taken together, the strategy that bacterial metabolism enables in situ and simultaneous generations of ·OH and photothermal agent Cu_2_S for PTT-enhanced nanocatalytic tumor therapy is conceptually feasible.

### Colonization of Cu_2_O@ΔSt in Tumor-Bearing Mice and Healthy Mice

**Fig. 2:**
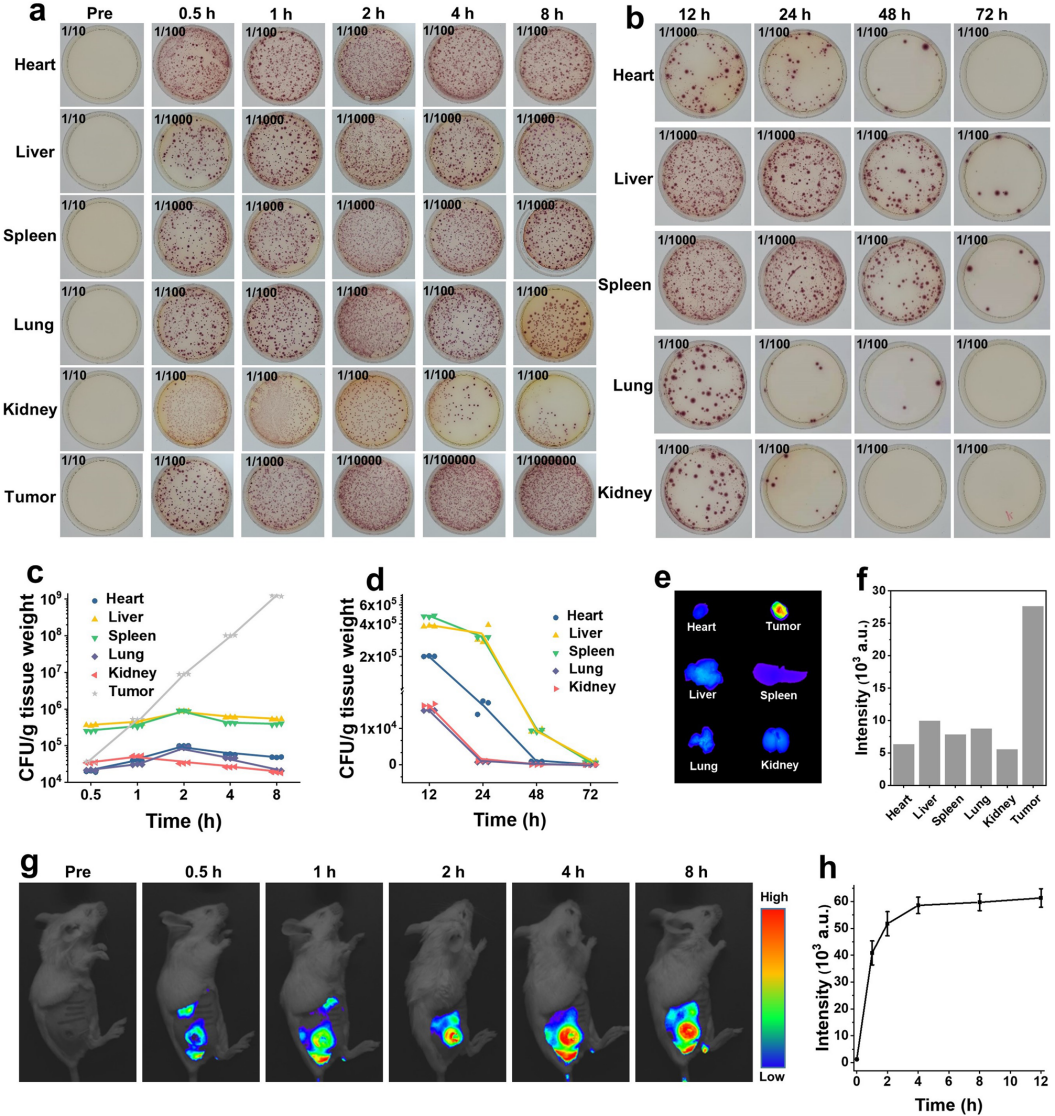
In vivo distributions of Cu_2_O@ΔSt. **a, b** Representative photographs of chromogenic Luria–Bertani (LB) agar plates and **c, d** quantifications of bacterial colonization in different organs collected from **a, c** CT26-bearing mice or **b, d** healthy mice at varied time durations after the injections of bacteria (*n* = 3, mean ± standard error). **e** Fluorescence imaging and **f** corresponding quantifications of fluorescence intensities of main organs and tumors isolated from mice in 8-h post-injection of IR780-labeled Cu_2_O@ΔSt (*n* = 3, mean ± standard error). **g** In vivo fluorescence images of CT26 tumor-bearing mice and **h** corresponding fluorescence intensities at the tumor site over time after the injection of IR780 labeled Cu_2_O@ΔSt

It is essential to actively deliver Cu_2_O to the tumor site for the microbiotic nanomedicine Cu_2_O@ΔSt to perform photothermal-enhanced nanocatalytic tumor therapy in vivo. Therefore, the behaviors of Cu_2_O@ΔSt in mice were systematically investigated in the following. Female CT26 subcutaneous tumor-bearing BABL/C mice were injected intravenously with Cu_2_O@ΔSt at a dose of 5 × 10^5^ CFU per mouse and then sacrificed in 0.5, 1-, 2-, 4-, and 8-h post-injection. Then, the collected main organs and tumors of mice were homogenized, serially diluted, and spread on chromogenic Luria–Bertani (LB) agar plates for incubation for 24 h. By counting the CFU number of bacteria in each plate, it can be seen that the CFU number of bacteria in the major organs such as heart, liver, spleen, kidney, and lung decreases gradually over time. In marked contrast, the CFUnumber in the tumor site increases exponentially over time (Fig. 2a, c). This phenomenon suggests that ΔSt could colonize efficiently in the hypoxic, immunosuppressed, and biochemically unique tumor microenvironment while being gradually eliminated in other normal tissues, which ensures tumor-specific therapy based on Cu_2_O@ΔSt. The long-term behaviors of Cu_2_O@ΔSt in normal organs of healthy mice were also explored by counting the live bacteria numbers in the major organs of healthy mice after intravenous injection of Cu_2_O@ΔSt (5 × 10^5^ CFU per mouse) at the given time points. As shown in Fig. 2b, d, at early time points (e.g., 12 and 24 h), the injected Cu_2_O@ΔSt shows significant accumulations mainly in the liver and spleen and presents rapidly decreased CFU values in all examined organs. In 3 days, almost no bacterial infection can be found in the major organs of the mice, probably due to the effective clearance of bacteria from the normal organs of the mice by the immune system. To visualize the tumor-targeting performance of Cu_2_O@ΔSt in vivo, the IR780-labeled bacteria was intravenously injected into CT26 tumor-bearing Balb/c mice, and then the fluorescence distribution in mice at scheduled time points was detected by a small animal fluorescence imaging system. We observed that the fluorescence intensity of Cu_2_O@ΔSt at the tumor site gradually intensified over time and reached a high-intensity plateau 4-h post-injection (Fig. 2g, h), demonstrating the excellent tumor-targeting performance of Cu_2_O@ΔSt. Such an attractive targeting capability of Cu_2_O@ΔSt was also demonstrated by ex vivo fluorescence imaging of tumors and other organs (Fig. 2e, f).

### In Vitro ICD Production and Activation of Immune Cells (DC Cells)

**Fig. 3:**
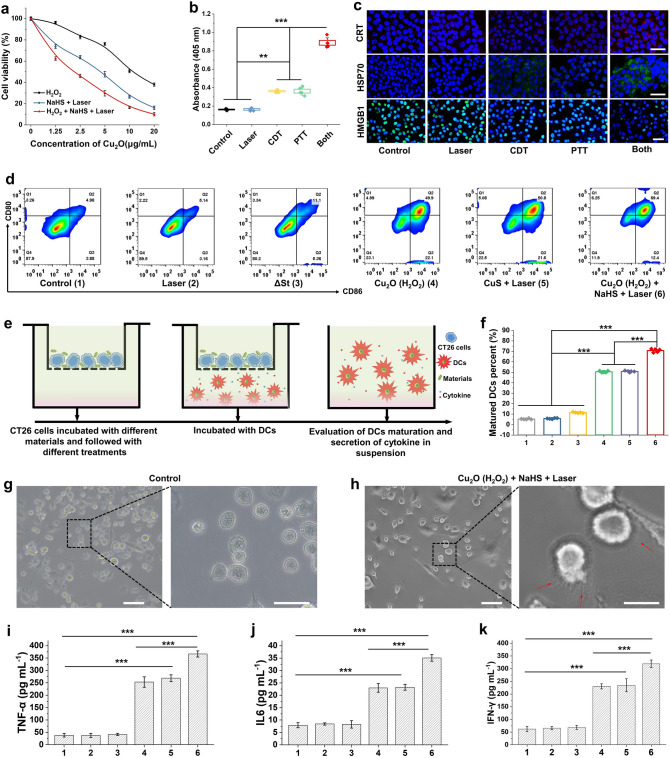
Cytotoxic and in vitro DCs maturation study. **a** Viability of CT26 cells after different treatments. **b** Caspase-3 activity in CT26 cells after different treatments (*n* = 6, mean ± standard error). **c** Immunofluorescence images of CRT, HSP70, and HMGB1 of CT26 cells received different treatments (scale bar, 50 μm). **d** Flow cytometry results of DCs after different treatments in the transwell system. **e** Schematic illustration of the transwell system experiment. DCs were cultured in the lower chamber, while pretreated CT26 cells were placed in the upper chamber. **f** Quantifications of the percentages of mature DCs (CD11c^+^CD80^+^CD86.^+^) (*n* = 5, mean ± standard error). **g, h** Photo-microscopy image of DCs after different treatments, scale bar: left, 100 μm; right, 40 μm. **i, k** Levels of TNF-α, IL6, and IFN-γ in DCs suspensions which were measured by ELISA (*n* = 5, mean ± standard error)

To exclude the effect of direct interaction between tumor cells and bacteria, we used Cu_2_O-mediated nanocatalytic therapy and CuS-mediated PTT to mimic the anticancer effects of Cu_2_O@ΔSt in vitro. The standard cell counting kit-8 (CCK-8) assays were then conducted to explore the influence of different treatments on tumor cell viability in vitro. As shown in Fig. 3a, both nanocatalytic therapy (Cu_2_O + H_2_O_2_) and PTT (Cu_2_O + NaHS + laser) behavior significant concentration-dependent effects on cell viability, and the combined treatment (Cu_2_O + H_2_O_2_ + NaHS + laser) exhibits much stronger cancer cell-killing efficacy. Under the continuous attack by intracellularly generated hydroxyl radicals and the thermal effect of laser irradiation, much-enhanced expression levels of apoptosis proteins (caspase-3, Fig. 3b) in treated tumor cells have been detected, indicating the marked apoptosis of tumor cells. To obtain more reliable evidence, the calcein acetoxymethyl ester (Calcein-AM)/PI (propidium iodide) staining assay was conducted to visualize the living and dead cells via green and red fluorescence emissions, respectively. It can be seen from Fig. S6 that only bright red PI fluorescence strongly presents in the combined treated group, while both green and red fluorescences are visible in either Cu_2_O + H_2_O_2_ or Cu_2_O + NaHS + laser group, indicating that the CuS-mediated photothermal effect could effectively amplify the Cu_2_O-triggered nanocatalytic cancer-killing efficacy in vitro. Recent studies showed that nanocatalytic therapy-derived ROS-mediated tumor damage could trigger immunogenetic cell death (ICD) cascade to elevate tumor sensitivity toward immunotherapy, and such a process could be further amplified by PTT [33]. Therefore, the efficacies of different treatments in inducing tumoral ICD were then evaluated in vitro. As two hallmark events in ICD, the expressions of CRT and HSP70, as well as the release of HMGB1 in cancer cells after different treatments, were monitored. Immunofluorescence images reveal (Fig. 3c) that the fluorescence intensities of CRT and HSP70 in the combined treatment group are stronger than those in single nanocatalytic treatment or PTT. The expressions of CRT and HSP70 in cancer cells of control and laser groups are negligible, and the fluorescence intensity loss of HMGB1 is greater in the combinational treatment group than that in the single treatment groups, suggesting that the combination treatment has induced more release of HMGB1 from cell nuclei. All of these results suggest that the simulated photothermal-enhanced nanocatalytic treatment is capable of triggering large-scale tumoral ICD, which is conducive to boosting the autologous immune system to fight cancer.

Dendritic cells play an important role in the initiation, regulation, and maintenance of immune responses [34]. Tumor cells possessing high immunogenicity can rapidly trigger the maturation of DC cells to further activate the adaptive immune response. The freshly isolated bone marrow dendritic cells (BMDCs) were co-cultured with pretreated CT26 cells and then stained for flow cytometric analysis to evaluate the capability of tumor cells in inducing the maturation of DCs by different treatments (Fig. 3e). Compared with cancer cells pretreated by laser- or bacteria-only, these pretreated by both nanocatalytic therapy (Cu_2_O + H_2_O_2_) and PTT (Cu_2_O + NaHS + laser) are much more effective in promoting the maturation of DCs, where the percentages of mature DCs (CD11c^+^CD80^+^CD86^+^ cells) reach 49.9% and 50.8% after coincubation, respectively (Fig. 3d, f). Noticeably, the tumor cells by the combinational treatment trigger the highest level of maturation of DCs (69.4%) due to the highest release levels of DAMPs and tumor-associated antigens (TAAs). More intuitively, the immature DCs present a large number of synapses on their surface in 24 h of incubation with combination-treated tumor cells, indicating their elevated maturation, in comparison with the controls (Fig. 3g, h). Additionally, as one of the typical indicators of the immune responses of DCs, the levels of DCs-secreted immune cytokines were also quantitatively measured by ELISA assay. Consistent with the flow cytometry analysis, the DCs secreted the highest levels of immune cytokines, such as TNF-α, IL-6, and IFN-γ, upon the stimulation by tumor cells treated with photothermal-enhanced nanocatalytic therapy, compared to tumor cells pretreated with other treatments (Fig. 3i, k). These results demonstrate that the strategy of photothermal-enhanced nanocatalytic therapy under NIR irradiation holds great promise for triggering ICD of tumor cells and thus promoting related immune cell infiltration in tumor tissues.

### Bacterial Metabolism-Initiated and Photothermal-Enhanced Nanocatalytic Therapy

Before conducting in vivo tumor therapy experiments, the safe dose of bacteria was initially determined by observing the change in body weight of healthy mice after the injections of different doses of the bacteria. It can be seen from Fig. S7a that the excessive bacterial dose (10^6^ CFU Cu_2_OΔ@St) has caused a significant loss of body weight in the mice, while the lower doses are tolerable for the mice. Therefore, we chose the dose of 10^5^ CFU Cu_2_OΔ@St for the subsequent in vivo tests. After the injection of 10^5^ CFU Cu_2_OΔ@St at different time points, blood was collected from mice for hematological examination to further demonstrate the safety of this injected dose. Compared with the control group, no significant difference in the biochemical indexes such as blood, liver, and kidney function of the mice was recorded in the experimental group (Fig. S7b–l). The main organs of mice were harvested for hematoxylin and eosin (H&E) staining after 30 days of treatment, wherein no obvious necrosis or inflammation was observed (Fig. S8). Additionally, the result of the hemolysis assay demonstrated that Cu_2_O@ΔSt at a dose of 10^5^ CFU did not induce blood hemolysis in mice, further indicating their excellent biosafety (Fig. S9). All of these results indicate that Cu_2_O@ΔSt possesses favorable biosafety and biocompatibility at the used dose of 10^5^ CFU.

**Fig. 4:**
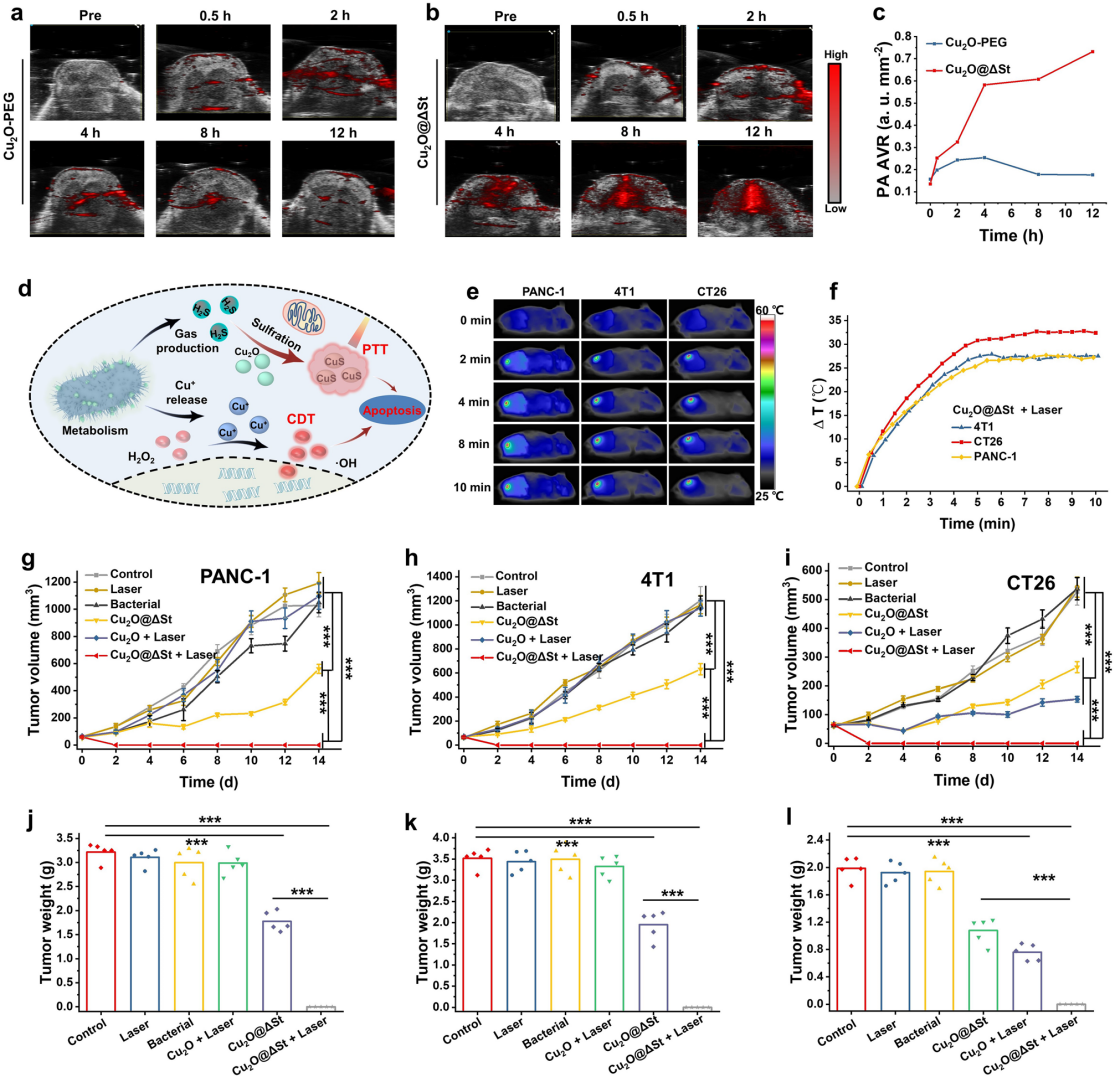
In vivo tumor therapeutic effect of Cu_2_O@ΔSt on 4T1 on different tumor-bearing mice models. **a, b** PA images of CT26 tumor-bearing mice after intravenous injections of PEG-Cu_2_O NPs only and Cu_2_O@ΔSt, and **c** corresponding PA signal intensities at tumor sites. **d** Schematic illustration of the mechanism of Cu_2_O@ΔSt-triggered therapeutic effect. **e** IR images at different time intervals of mice after injections of Cu_2_O@ΔSt. **f** The corresponding temperature elevations over time of mice after different treatments. The tumor volumes and weights of different types of tumor-bearing mice at the ends of various treatments, including **g, j** PANC-1 tumor-bearing mice model, **h, k** 4T1 tumor-bearing mice model, and **i, l** CT26 tumor-bearing mice model (*n* = 5, mean ± standard error)

The excellent biocompatibility of the Cu_2_O@ΔSt prompted us to explore its capability to trigger the bacteria metabolism-initiated and photothermal-enhanced nanocatalytic therapy for various types of cancer. As Fig. 4d displays, the in situ sulfuration of Cu_2_O by bacterial metabolism-derived H_2_S forming the CuS photothermal agent is the key to achieving photothermal-enhanced nanocatalytic cancer therapy under NIR irradiation. To investigate the vascular disruption and coagulation in tumors of mice caused by Cu_2_OΔ@St, which may affect the absorption of NIR of tumors, we directly measured the hemoglobin content in tumors of mice collected at different time points post-injection of the bacteria (10^5^ CFU). It can be found that the hemoglobin content of the tumor site in mice did not change significantly after the injection of bacteria, and it can be predicted that low doses of bacteria would not induce alterations in the absorbing properties of tumor tissues (Fig. S10). It has been demonstrated that the CuS is an excellent photoacoustic (PA) imaging agent owing to its strong NIR absorption, while Cu_2_O-PEG presents a weak PA signal [24]. Therefore, if a significant PA signal can be detected after the injection of Cu_2_O@ΔSt in CT26 tumor-bearing mice, the in situ formation of CuS species through the sulfuration of Cu_2_O by H_2_S in vivo can be confirmed. Herein, the CT26 tumor-bearing mice model was used, and the PA images of mice were collected at different time points after the intravenous injections of PEG-Cu_2_O NPs and Cu_2_O@ΔSt. In mice injected with free Cu_2_O-PEG, a certain PA signal was detected at the tumor site in 2 h after injection, which may be due to the certain extent of Cu_2_O sulfuration by overexpressed H_2_S in CT26 tumor tissue (Fig. S11). However, along with the gradual excretion of small PEG-Cu_2_O NPs from mice, the intensity of the PA signal at the tumor site decreased significantly (Fig. 4a). In striking contrast, the PA signals at the tumor site of mice were significantly enhanced overtime after the injection of Cu_2_O@ΔSt, owing to the continuous sulfuration of Cu_2_O by ΔSt metabolism-generated H_2_S (Fig. 4b, c). Additionally, in 12 h of injection, the H_2_S concentrations in the blood, major organs, and tumor sites of mice were further measured by using the standard H_2_S detection kit assay. The measured H_2_S concentration in the tumor tissue of mice injected with Cu_2_O@ΔSt is 10 times higher than that with Cu_2_O, whereas no significant difference in the H_2_S concentration in blood and other organs has been found, attributing to the tumor-specific H_2_S production property of ΔSt (Fig. S12). More importantly, the Cu^+^ released from Cu_2_O in exacerbated acidic TME has triggered the Fenton-like reaction to generate abundant ·OH as evidenced by the in vivo ROS staining assay (Fig. S13). The result shows that the intratumoral ROS level of mice in the Cu_2_O@ΔSt-treated group was largely elevated compared with the mice in the PEG-Cu_2_O NPs-treated group. Therefore, it can be reasonably inferred that after intratumoral colonization, the microbial nanomedicine Cu_2_O@ΔSt has produced free Cu^+^ species as the Fonten-like agent and CuS species as the photothermal agent by bacterial metabolism-generated H_2_S within the tumors, further enabling photothermal-enhanced nanocatalytic tumor therapy under NIR laser irradiation.

To evaluate the antitumoral efficacy via the above-revealed photothermal-enhanced nanocatalytic tumor therapy, we first investigated the in vivo photothermal performance of Cu_2_O@ΔSt on various types of tumors-bearing mice (PANC-1, 4T1, and CT26 tumor). The photothermal heating of those tumors was achieved by irradiating these tumors with an 808 nm laser at a power density of 1.5 W cm^−2^ for 10 min in 12 h of Cu_2_O@ΔSt injection, which all presented rapid laser-induced temperature increases (Fig. 4e, f), in contrast to the negligible heating effect of saline (Fig. S14). Based on the ROS generating performance of Cu_2_O@ΔSt and its apparent heating effect respectively due to the Cu^+^ and CuS formations under laser irradiation, we further investigated its tumor growth inhibitory effect on the above three tumor-bearing mice models under laser irradiation. The body weights and average tumor volumes were recorded for mice after different treatments, including (1) PBS, (2) laser irradiation, (3) ΔSt injection, (4) Cu_2_O@ΔSt injection, (5) Cu_2_O-PEG (20 mg kg^−1^) injection + laser, and (6) Cu_2_O@ΔSt injection + laser. No significant body weight losses were found in the mice of all experimental groups compared to these of control group, suggesting that these therapeutic parameters are well-tolerable for mice (Fig. S15). As displayed by the tumor growth curves and final tumor weights (Fig. 4g–l), the tumors volumes of three types of tumor-bearing mice in groups (1), (2), and (3) have increased rapidly, while the Cu_2_O@ΔSt group shows a certain degree of inhibition effect of tumor growth owing to the generated ROS. Notably, Cu_2_O-PEG + laser treatment has produced a significant tumor growth inhibition effect only on CT26 tumor-bearing mice, contributed by the formation of a certain amount of CuS by the in situ reaction between Cu_2_O and endogenous H_2_S. Encouragingly, after Cu_2_O@ΔSt + laser irradiation treatment, complete tumor ablation with tolerable whole-body toxicity has been achieved by such bacteria metabolism-initiated and photothermal-enhanced nanocatalytic therapy for all three types of tumor models.

### Activation of Anticancer Immunity by Cu_2_O@ΔSt

**Fig. 5:**
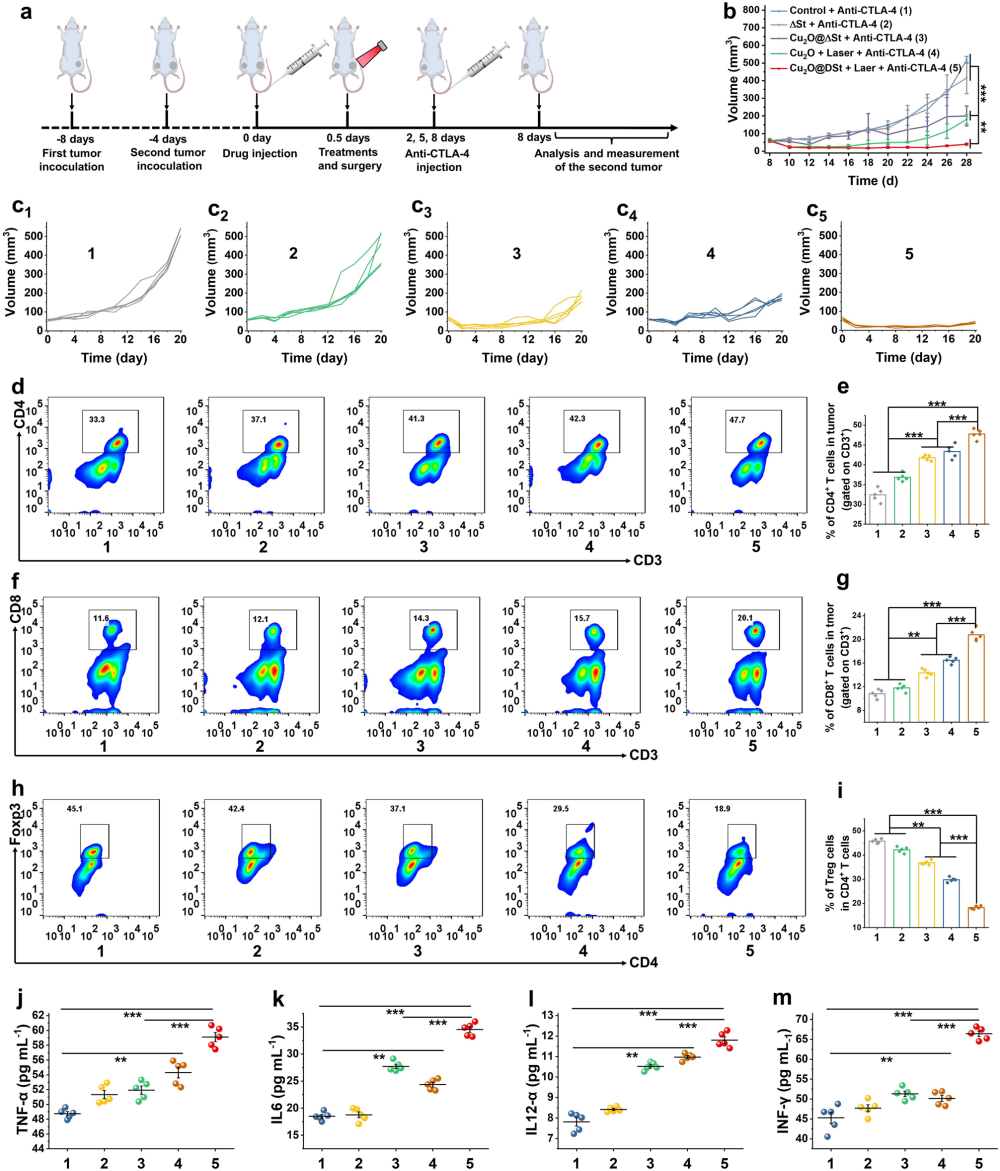
Anticancer activity of Cu_2_O@ΔSt plus anti-CTLA-4 treatment in murine colorectal cancer models. **a** Schematic illustration of CT26 tumor-bearing mouse model establishment and treatment procedures in vivo. **b** Average volume changes of the tumor on the contralateral side of mice in different groups (*n* = 5, mean ± standard error). **c** Volume change curves of contralateral tumor of each mouse after various treatments. **d, f** Flow cytometry analyses and **e, g** corresponding quantifications of CD4^+^/CD8 + T cells (gated on CD3^+^ T cells) (*n* = 5, mean ± standard error). **h** FCM analysis and **i** corresponding quantification of FoxP3^+^/CD4^+^ regulatory T (Treg) cells among CD4.^+^ cells. Secretion levels of **j** TNF-α, **k** IL-6, **l** IL12, and **m** IFN-γ in serum(*n* = 5, mean ± standard error)

It has been demonstrated that certain modalities of tumor therapeutics, such as PTT and ROS-based dynamic therapies, can trigger strong tumoral ICD, thereby activating dendritic cells (DCs) and further augmenting the infiltration of tumoricidal T cells to evoke robust antitumoral immuno-responses. Therefore, we then studied the in vivo ICD triggering performance by such a bacterial metabolism-initiated and photothermal-enhanced nanocatalytic therapy. The tumors were collected and sliced to detect the expressions of CRT and HSP70, as well as the release of HMGB1 in tumor tissues after different treatments. As immunofluorescence images shows (Figs. 5a and S16), the two hallmark events in ICD, the upregulated expressions of CRT and HSP70, as well as the release of HMGB1 in cancer cells, can be evidenced in the tumor tissues, indicating that a large-scale ICD in tumor tissues has been induced after the Cu_2_O@ΔSt plus laser irradiation treatment. The Western Blot assays further confirm the effectiveness of Cu_2_O@ΔSt in triggering ICD under laser irradiation (Fig. S17). Once expressed on the surface of tumor cells, CRT proteins present an “eat me” signal to recruit DCs to engulf the corpses and debris of dying tumor cells. Simultaneously, the released HMGB1 acts as a natural adjuvant to stimulate the maturation of DCs, which then present TAAs to T cells. To confirm the DCs maturation in vivo, flow cytometry analyses were then conducted, (Fig. 5b, d), which show much higher proportions of mature DCs in ipsilateral inguinal lymph nodes nearby tumors in (4) and (5) groups than that in the (1), (2), (3) group, while the proportion of mature DCs in the (6) group is the highest. More importantly, the significant infiltration of CD8^+^ and CD4^+^ T cells in the tumors of mice was determined owing to the effective DCs-mediated presentation of TAAs to T cells upon Cu_2_O@ΔSt + laser treatment (Fig. 5c, e, and f). It has also been found that Cu_2_O@ΔSt + laser treatment significantly elevates the concentration of cytokines (such as TNF-α, IL-6, and IFN-γ) in mouse serum that favorably triggers antitumor immune responses (Fig. 5g-i). Thus, the tumor debris after bacterial metabolism-initiated and photothermal-enhanced nanocatalytic therapy is capable of eliciting powerful immune responses, which are tumor-specific and able to provide abscopal therapeutic effects against distant and/or metastatic tumors not accessible by laser irradiation.

### Bacterial Metabolism-Initiated and Photothermal-Enhanced Nanocatalytic Immunotherapy

**Fig. 6:**
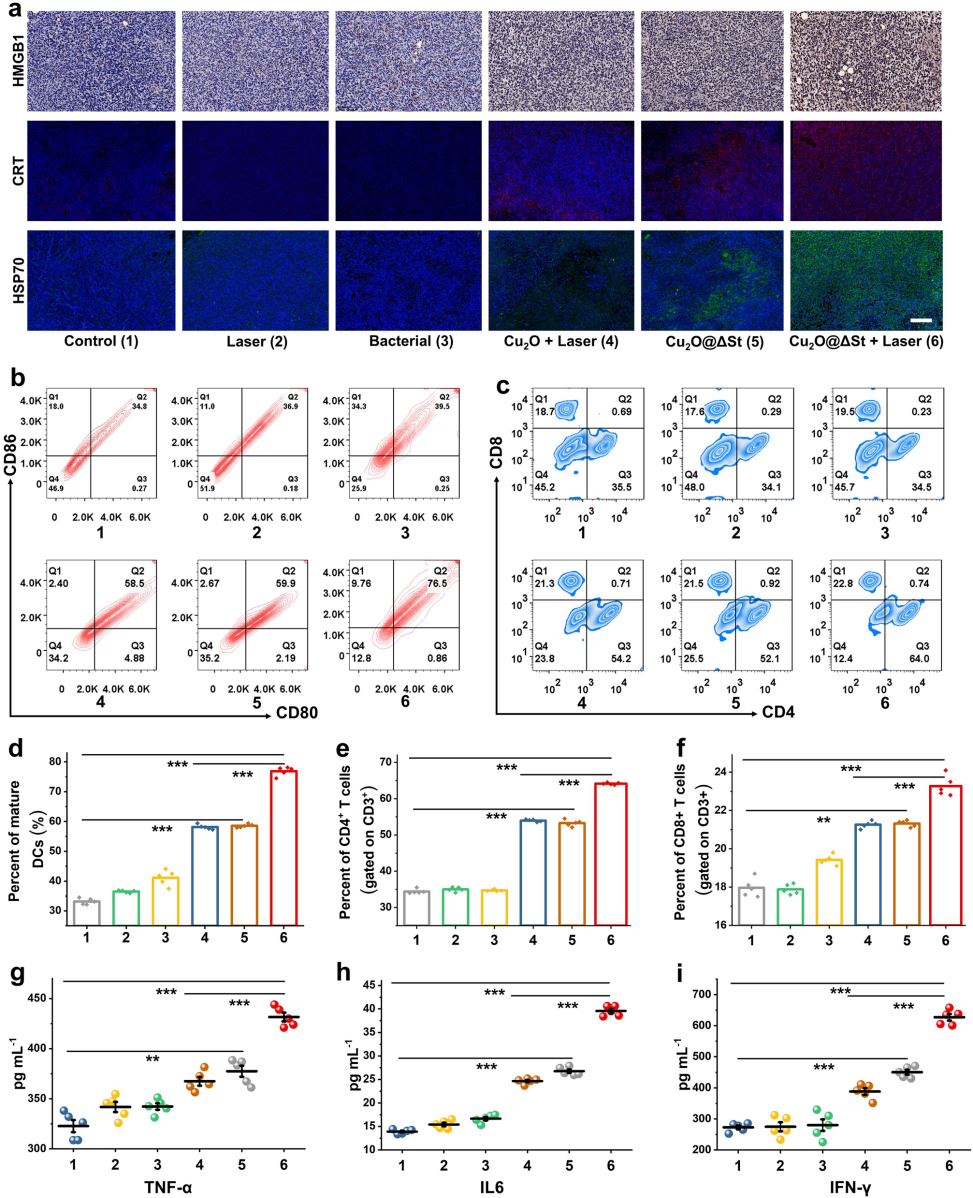
Immune activation evaluation in vivo. **a** Immunofluorescence analysis of specific protein expressions after different treatments (HMGB1, CRT, and HSP70) from tumor tissue (scale bar, 100 μm). **b** Flow cytometry analysis and **d** quantification results of in vivo mature DCs in tumors of mice after different treatments (gated on CD11c^+^ DC cells). **c** Flow cytometry analysis and **e, f** quantification results of CD4^+^/CD8^+^ T cells (gated on CD3.^+^ T cells) (*n* = 5, mean ± standard error). The contents of **g** IL-6, **h** TNF-α, and **i** IFN-γ in tumor tissues of mice after different treatments measured by ELISA (*n* = 5, mean ± standard error)

Next, the abscopal therapeutic effect of Cu_2_O@ΔSt on distant tumors was evaluated on a bilaterally CT26 tumor-bearing mice model. In this assay, the antibody against CTLA-4 (anti-CTLA-4), which has been approved by the FDA, was employed to further augment the antitumor immunity of mice by inhibiting the activities of immune-suppressive regulatory T (Treg) cells. As shown in Fig. 6a, the primary tumor was removed by five different treatments, including surgery (groups 1), bacteria plus surgery (groups 2), Cu_2_O@ΔSt plus surgery (groups 3), Cu_2_O + laser plus surgery (groups 4), and Cu_2_O@ΔSt + laser (groups 5). After the removals of the primary tumors, mice were injected with anti-CTLA-4 on days 2, 5, and 8 at a dose of 10 μg per mouse, and the volumes of distant tumors were monitored afterward (Fig. 6b, c). Compared to the mice with surgical removal of their primary tumors (group 1), the distant tumors of mice in groups 3 and 4 were significantly suppressed in combination with anti-CTLA-4 treatment, owing to the certain activation of the antitumor immune response in mice by single nanocatalytic therapy or PTT of primary tumors and the Cu_2_O-mediated nanocatalytic therapy effect on distant tumors. Notably, the most dramatic abscopal therapeutic effect was observed for the combined treatment of bacteria-initiated and photothermal-enhanced nanocatalytic therapy with anti-CTLA-4 administration (group 5), where distant tumor growths were almost completely suppressed following their primary tumor eradication by Cu_2_O@ΔSt + laser treatment. The combined bacteria and anti-CTLA-4 administrations (group 2) appear to be less effective in suppressing the distant tumors than group 5, due to the lower immune stimulation effect of such a combination therapy, which indicates that the immune stimulation effect by the debris of ablated primary tumors is responsible for the induced abscopal antitumor immune responses. Additionally, no significant lung metastases of tumor cells have been observed in mice with their primary tumors being ablated by Cu_2_O@ΔSt + laser (Fig. S18), suggesting that the above-mentioned treatment strategy simultaneously presents a favorable antitumoral metastasis efficacy.

To understand the underlying immune mechanism of distant tumor suppression and antitumoral metastasis, immune cells in the mimic distant tumors and the concentrations of immune cytokines in serum (TNF-α, IL-6, IL12, and IFN-γ) were explored on the 11th day after the first treatment. It was found that the percentage of CD3^+^CD4^+^/CD3^+^CD8^+^ T cells representing cytotoxic T lymphocytes (CTLs) displayed the most significant increase in group 5 which received the combined Cu_2_O@ΔSt + laser and anti-CTLA-4 therapy (Fig. 6d, g). Meanwhile, as expected, the activities of immunosuppressive Treg cells (CD3^+^CD4^+^FoxP3^+^ T cells) were effectively inhibited by anti-CTLA-4 treatment (Fig. 6h, i). As an important indicator of antitumor immune equilibrium, the CTL/Treg ratio was calculated to be the highest in group 5 with the combined photothermal-enhanced nanocatalytic therapy and anti-CTLA-4 therapy, which is in accordance with tumor growth data (Fig. S19). In addition, the serum concentrations of several representative immune cytokines (TNF-α, IL-6, IL12, and IFN-γ) that play important roles in tumor immunotherapy in group 5 were also found to be the highest (Fig. 6j, k, l and m). Accordingly, the bacterial metabolism-initiated and photothermal-enhanced nanocatalytic therapy could effectively activate cellular immunity to suppress distant tumor growths and prevent tumor metastasis with the assistance of anti-CTLA-4.

### Long-term Immune Memory Effects

**Fig. 7:**
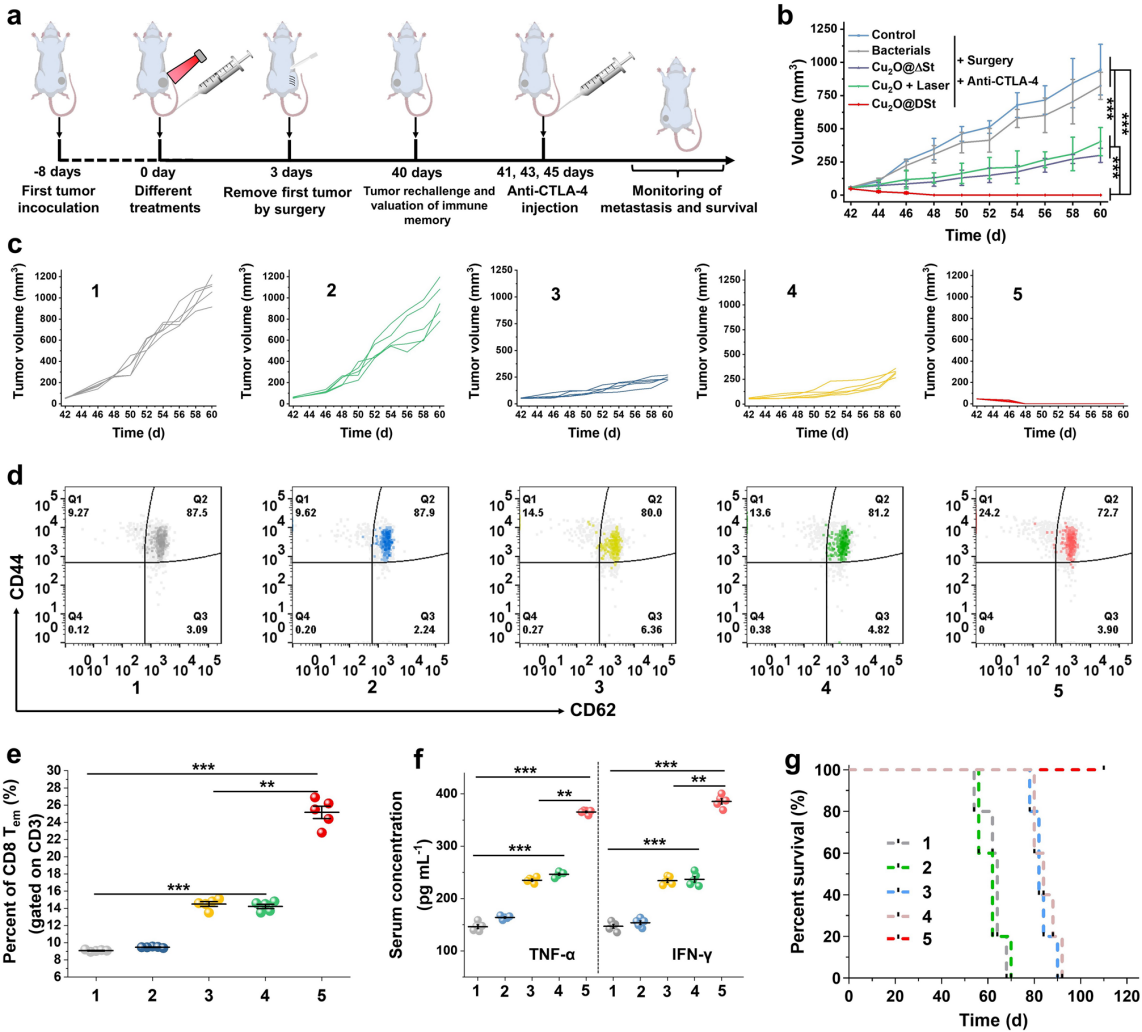
Long-term immune-memory effects of Cu_2_O@ΔSt. **a** Schematic illustration of Cu_2_O@ΔSt-based immunotherapy to inhibit cancer relapse. **b** Volume change curves of rechallenged tumors beyond 40 days after the elimination of their first tumors. **c** Volume change curves of rechallenged tumor of each mouse after various treatments. **d** Flow cytometry analysis and **e** corresponding quantification of the percentage of CD8^+^/CD3^+^ /CD44^+^/CD62^−^ effector memory T cells (T_EM_) gated on CD3^+^ cells in the spleen (spleens were collected on day 40 right before rechallenging mice with secondary tumors). **f** Cytokine levels of TNF-α and IFN-γ in sera from mice after different treatments. **g** Morbidity-free survival of different groups of mice after various treatments and subsequent rechallenge by CT26 tumors

As an important feature of adaptive immunities, the immune memory effect is capable of preventing the reoccurrence of the same disease over a long time period [35–37]. Therefore, we further investigated whether immune memory can be aroused by the bacterial metabolism-initiated and photothermal-enhanced nanocatalytic therapy of primary tumors or not. As displayed in the experimental procedure in Fig. 7a, in 3 days after different treatments, the first CT26 tumors were removed by surgery (groups 1, 2, 3, and 4), while tumors in group 5 were eradicated by Cu_2_O@ΔSt + laser treatment strategy. These mice were then rechallenged with a secondary inoculation of CT26-cancer cells on day 40, followed by the anti-CTLA-4 injections on days 41, 43, and 45. The volumes of secondary tumors and body weights of mice were then continuously recorded for 20 days (Figs. 7b, c and S20). For mice in groups 1 and 2 with the surgical removals of their first tumors, their second tumors exhibit rapid volume increases, even with the injection of anti-CTLA-4. Certain inhibition degrees on their rechallenged second tumor growth were observed in the mice of groups 3 and 4 due to the PTT or nanocatalytic therapy of the primary tumor which activated a certain level of immune memory effect. For mice with first tumors being removed by Cu_2_O@ΔSt + laser treatment, no appreciable growth of their rechallenged second tumors was observed (group 5) with the help of anti-CTLA-4. Moreover, all mice in group 5 survived post-tumor rechallenges for more than 110 days (Fig. 7g). Collectively, these results suggest that the Cu_2_O@ΔSt + laser treatment strategy produces a long-term immune memory effect, thereby protecting mice from the attack by rechallenged tumor cells after combined anti-CTLA-4 therapy. Finally, we probed the mechanism of the powerful immune memory post combined tumor immunotherapy by analyzing the related memory T cells and related cytokines. Both central memory T cells (T_CM_) and effector memory T cells (T_EM_) were collected from the spleen of mice for flow cytometric analysis on day 40 before the secondary inoculation of CT26-cancer cells. It has already been suggested that T_EM_ cells that can induce immediate immune protection by producing cytokines are mainly responsible for the immune memory effects [38]. From the flow cytometry data, it can be seen that the native T_CM_ cells had been obviously transformed into T_EM_ cells (CD3^+^CD8^+^CD44^+^CD62L^−^) in the spleen for mice with their primary tumors being eliminated by Cu_2_O@ΔSt + laser treatment (Fig. 7d, e). Furthermore, the mice with their first tumors being eradicated by bacterial metabolite-based CDT and PTT exhibit the most pronounced increases in serum levels of TNF-α and IFN-γ on day 47 compared to the mice in other groups (Fig. 7f), indicating that robust antitumor immune response has been induced upon rechallenging the mice with CT26 cells.

## Conclusion

In summary, we have constructed a novel microbiotic nanomedicine Cu_2_O@ΔSt by anchoring Cu_2_O NPs on the surface of engineered Salmonella typhimurium strain for achieving bacterial metabolism-initiated, photothermal-enhanced nanocatalytic immunotherapy. Due to the obligate hypoxia tropism of ΔSt, Cu_2_O@ΔSt could selectively colonize tumors after intravenous injection, thus preventing bacterial infections of healthy tissues. During metabolism within tumor, ΔSt is capable of specifically producing H_2_S gas and acidic substances via metabolism, and the generated H_2_S subsequently sulfidates the delivered Cu_2_O into CuS in situ, thus enabling the tumor-specific PTT upon local NIR laser irradiation. Simultaneously, the released Cu^+^ from Cu_2_O under acidified TME would catalyze the endogenous H_2_O_2_ decomposition to generate cytotoxic ·OH via Fenton-like reaction, thus performing nanocatalytic therapeutics. Importantly, the hydroxyl radical-mediated cellular damage could be further amplified by PTT upon irradiation by NIR laser, eliciting a large scale of intratumoral ICD. Such a bacterial metabolism-initiated photothermal-enhanced nanocatalytic therapy could result in massive apoptosis of tumor cells for tumor ablation, as successfully demonstrated on three types of tumor models. Thereafter, the released massive tumor antigens and DAPMS after the primary tumor ablation would trigger robust systemic antitumor immune responses to significantly inhibit both distant tumor growth and spontaneous tumor metastasis with the assistance of ICB therapy, which simultaneously offers a long-term immune memory effect to protect mice from the tumor cell rechallenge. Such a bacterial metabolism-initiated photothermal-enhanced nanocatalytic immunotherapy offers an effective paradigm for the establishment of novel and promising combinational cancer therapeutic modalities based on microbiotic nanomedicine.

## Supplementary Information

Below is the link to the electronic supplementary material.

Supplementary file1 (PDF 2114 KB)
