# Supplementary material for: Bacterial Metabolism-Initiated Nanocatalytic Tumor Immunotherapy
Source: Nanomicro Lett. 2022 Nov 11;14:220. doi: 10.1007/s40820-022-00951-0 (PMC9652197; doi:10.1007/s40820-022-00951-0)
Supplement: Supplementary file 1 — Supplementary file1 (PDF 2114 KB) [file 40820_2022_951_MOESM1_ESM.pdf]

Supporting Information for

Bacterial Metabolism-Initiated Nanocatalytic Tumor Immunotherapy

Wencheng Wu[1, #], Yinying Pu[2, #], Shuang Gao[3], Yucui Shen[3], Min Zhou[3, *], Heliang Yao[1], and Jianlin Shi[1, *]

[1] The State Key Lab of High Performance Ceramics and Superfine Microstructures, Shanghai Institute of Ceramics, Chinese Academy of Sciences; Research Unit of Shanghai Nanocatalytic Medicine in Specific Therapy for Serious Disease, Chinese Academy of Medical Sciences (2021RU012), Shanghai 200050, P. R. China

[2]Department of Medical Ultrasound, Shanghai Tenth People's Hospital, Ultrasound Research and Education Institute, Tongji University Cancer Center, Tongji University School of Medicine, Shanghai 200072, P. R. China

[3]Digestive endoscopy center, Shanghai Fourth People's Hospital to Tongji University, Shanghai 200081, P. R. China

[#] Wencheng Wu and Yinying Pu have contributed equally to this work.

*Corresponding authors. E-mail: jlshi@mail.sic.ac.cn (Jianlin Shi), crown_zhou@hotmail.com (Min Zhou)

Supplementary Figures

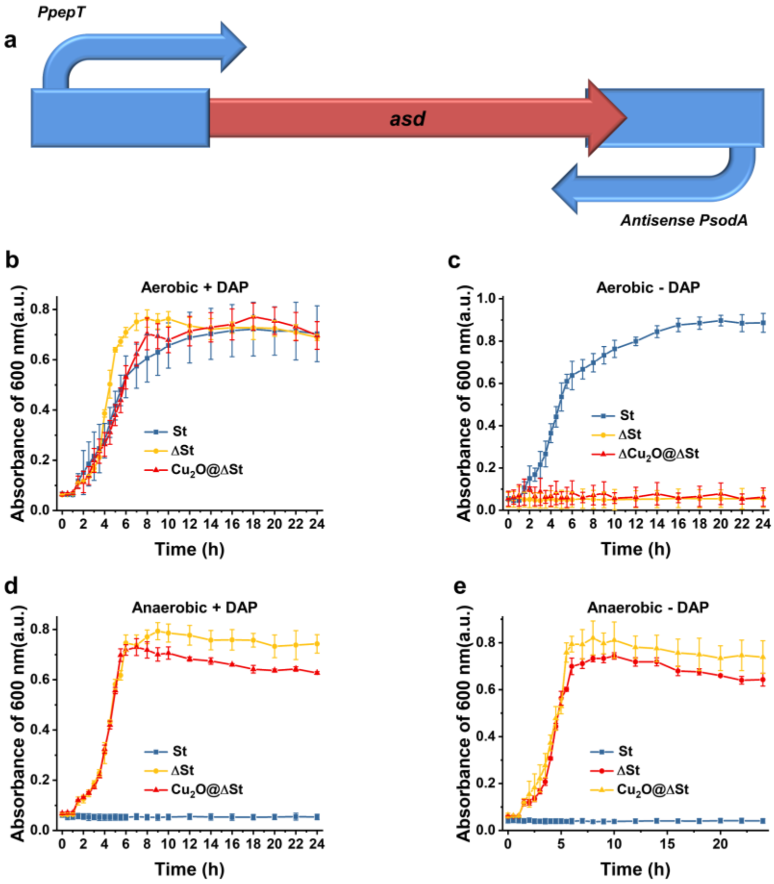

Fig. S1 (**a**) The fabrication of the asd gene expression cassette under hypoxic conditions. The sense promoter PpepT consists of the constitutive promoter P2 and the FNR-regulated promoter P1, and PsodA is the antisense promoter. (**b-e**) Growth rate of various strains under different conditions

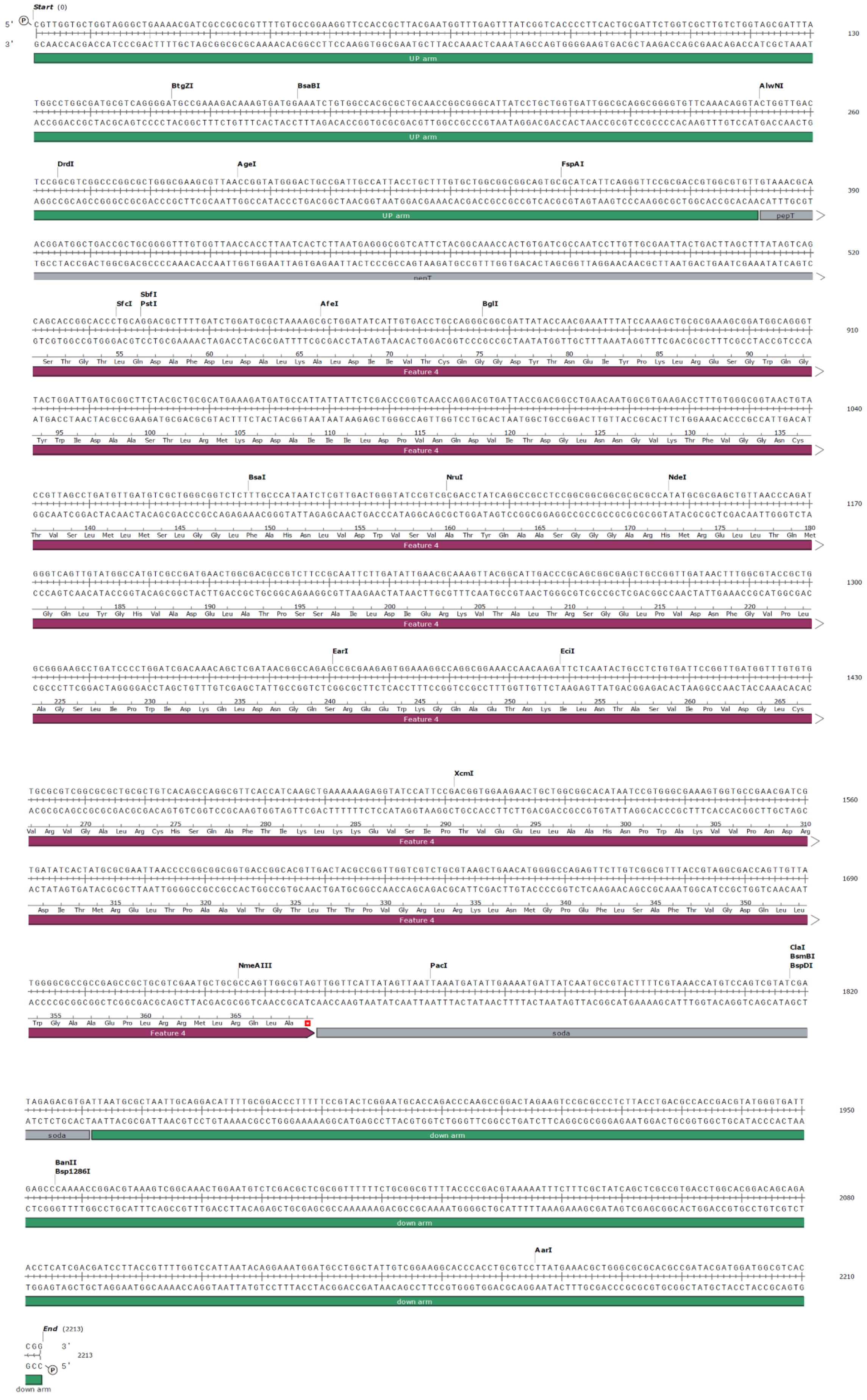

Fig. S2 Gene sequence map of engineered asd

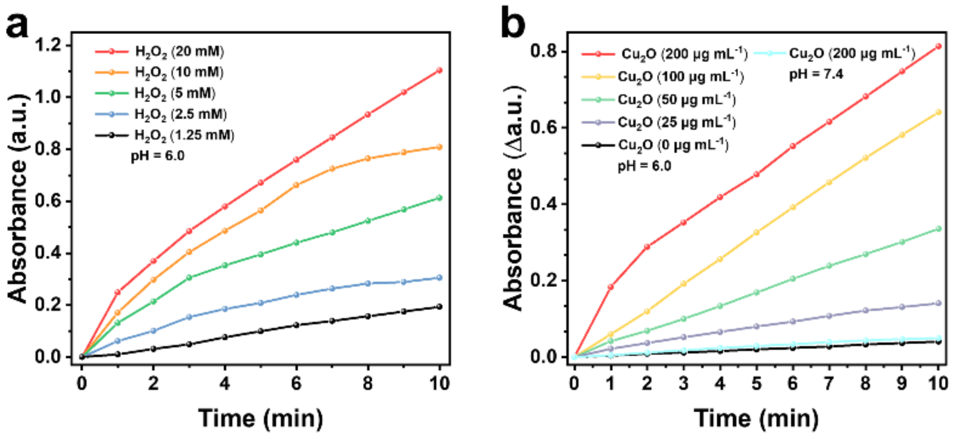

Fig. S3 The Cu_2O-mdiated production of ·OH as demonstrated by using 3,3',5,5'-tetramethylbenzidine (TMB), which can be oxidized by ·OH species to chromogenic TMB exhibiting characteristic absorption at 650 nm. As a result, PEG-Cu_2O NPs present catalyst concentration-, pH-, and substrate H_2O_2 concentration-dependent catalytic activity

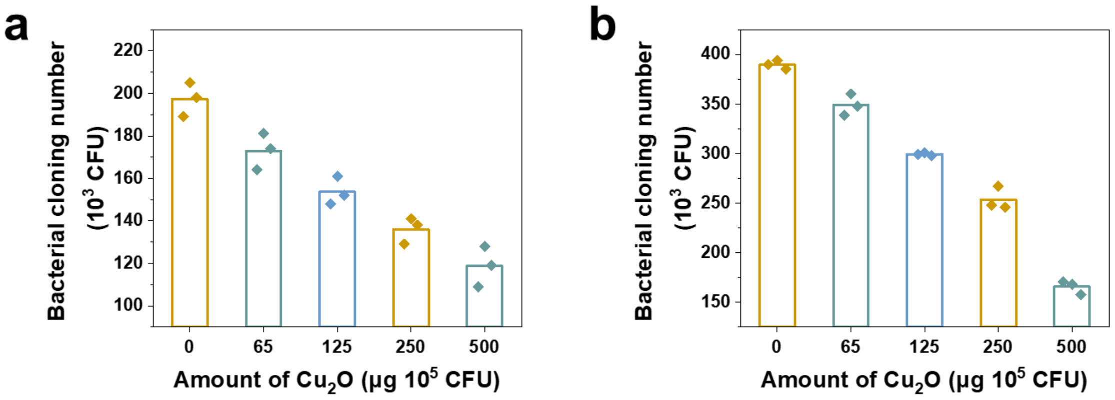

Fig. S4 Bacterial cloning number of ΔSt treated by different amount of PEG-Cu_2O for (**a**) 12 h and (**b**) 24 h

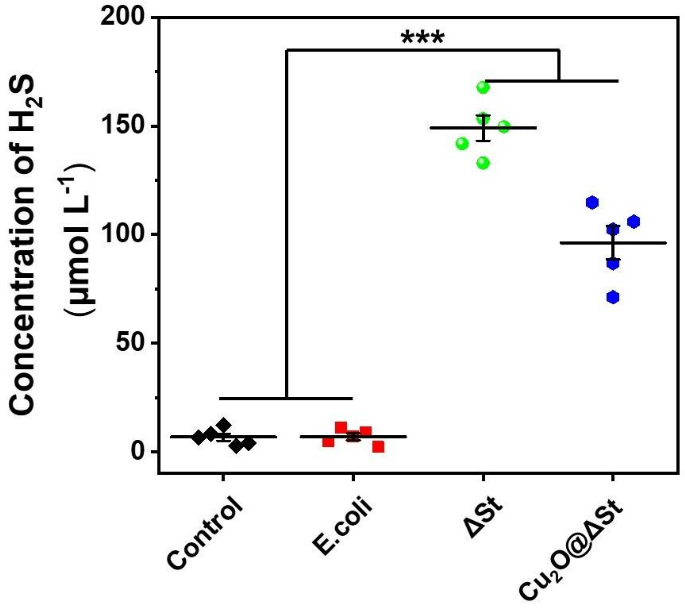

Fig. S5 The concentrations of dissolved H_2S in the medium in the presence of different strains of bacteria

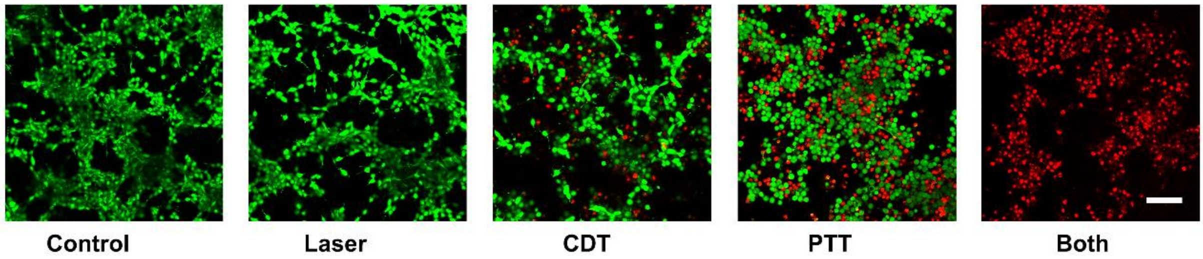

Fig. S6 CLSM images of 4T1 cells stained with Calcein-AM and PI after various treatments

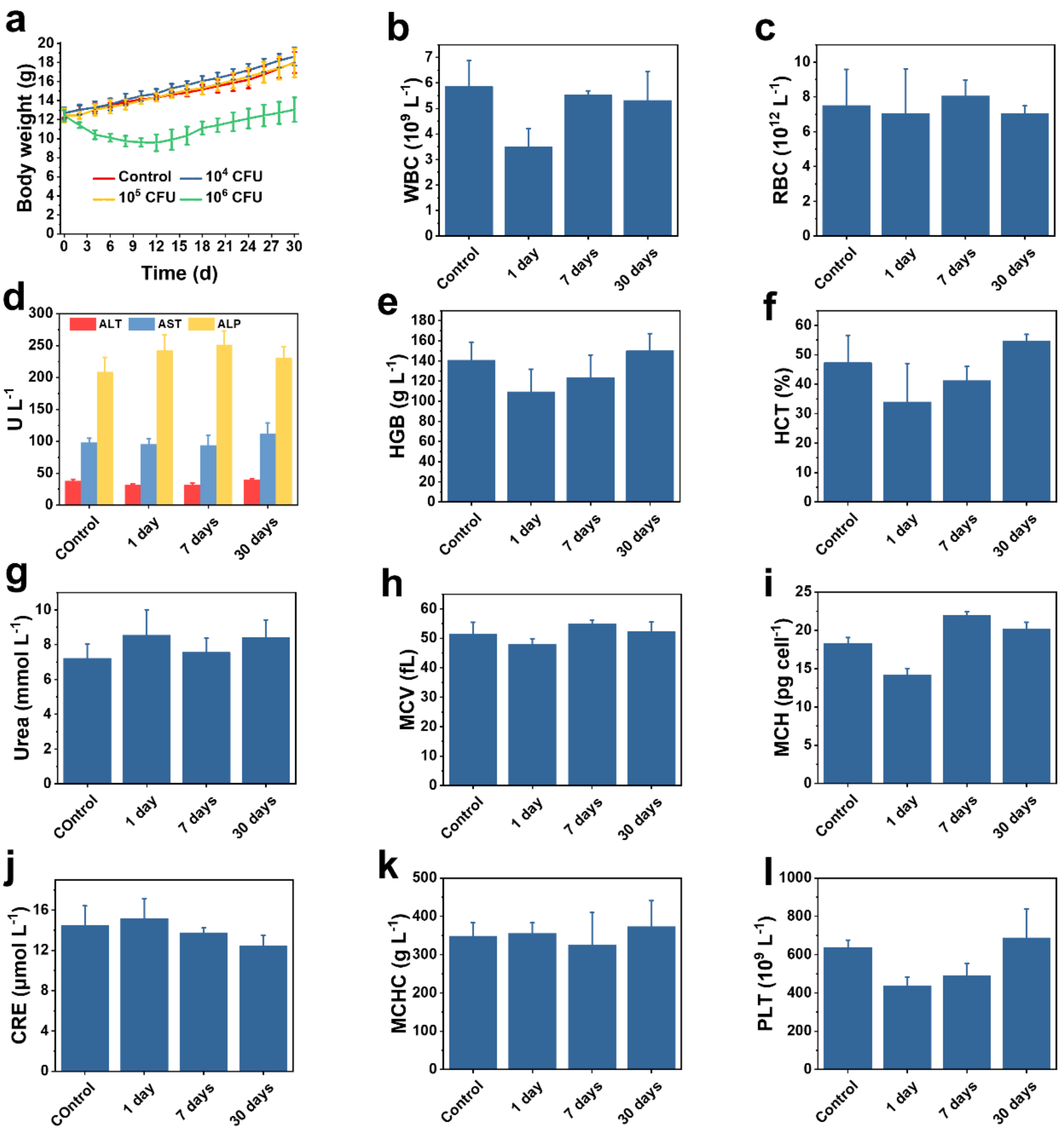

Fig. S7 (**a**) Body weight monitoring of mice during 30 days feeding post-injection of different doses of bacteria. (**b-l**) The hematological assays of mice at different time points after the injection of bacteria at the dose of 10^5 CFU

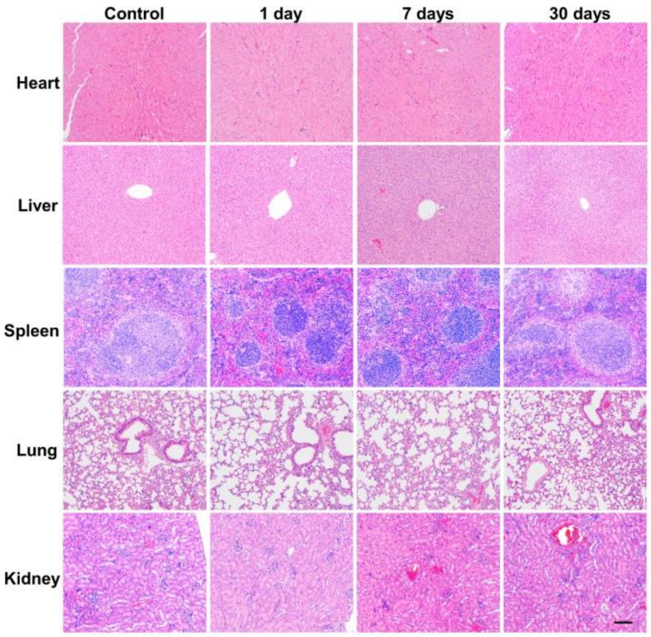

Fig. S8 H&E staining histological sections of main organs (heart, liver, spleen, lung, and kidney) obtained from the mice sacrificed at different time points, scale bar 200 μm

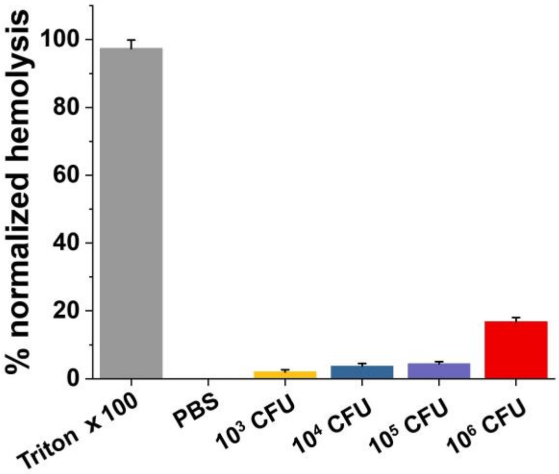

Fig. S9 Impact of Cu_2O@ΔSt on hemolysis after 1 h at different concentrations, in which Triton x100 (1%) and PBS were respectively used as positive and negative controls

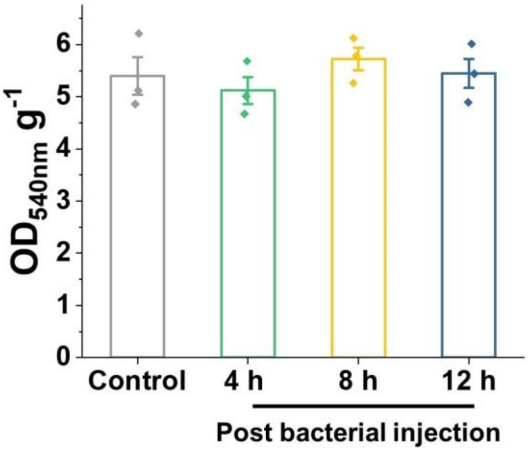

Fig. S10 Hemoglobin content in CT26 tumors at different time points after i.v. injection of Cu_2OΔ@St (10^5 CFU)

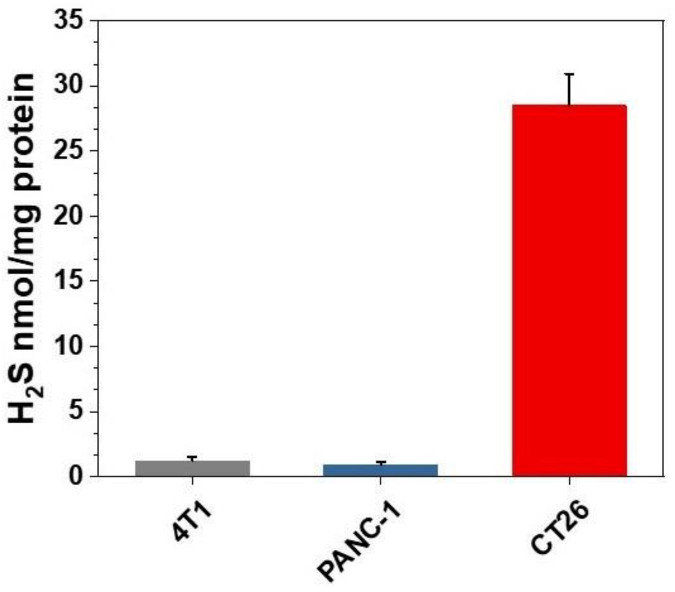

Fig. S11 H_2S concentration in the three types of tumor tissue

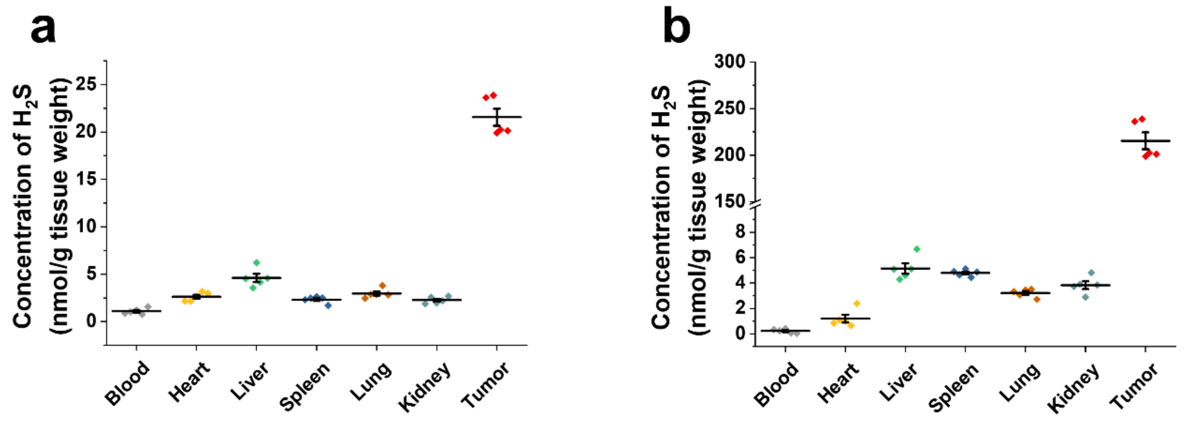

Fig. S12 Quantitative comparison of H_2S levels in different main organs and tumor tissues of mice after the injections of (**a**) PEG-Cu_2O and (**b**) Cu_2O@ΔSt

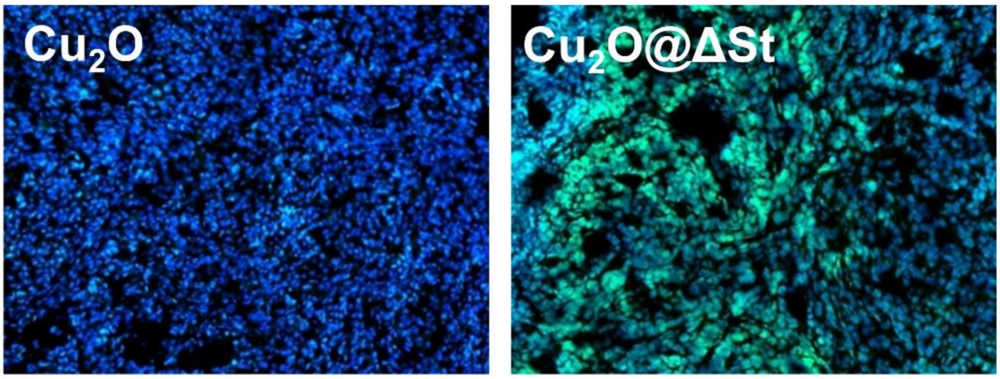

Fig. S13 DCFH-DA immunostaining of tumor sections after the injections of PEG-Cu_2O and Cu_2O@ΔSt

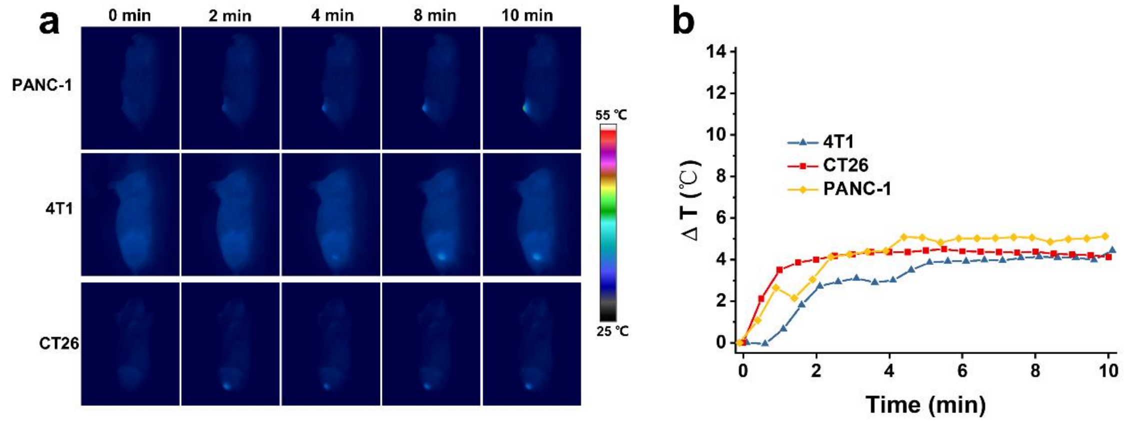

Fig. S14 (**a**) The IR images at different time intervals of mice after the injections of saline in mice bearing different types of tumor and (**b**) their corresponding temperature elevations at tumor site over time

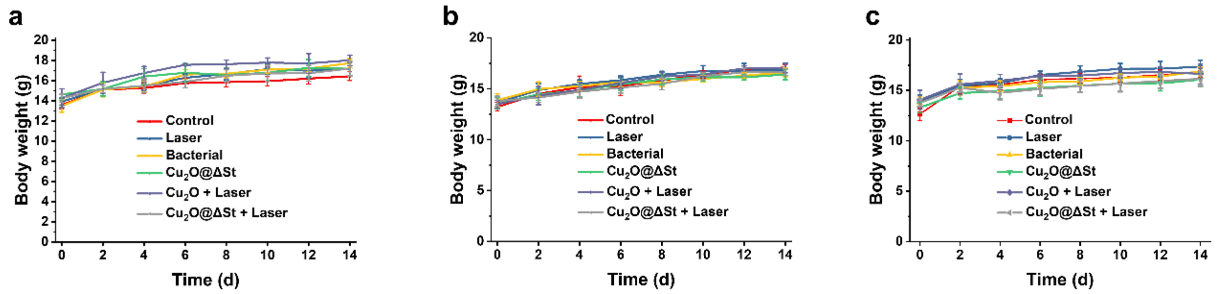

Fig. S15 Average body weights of different types of tumor-bearing mice after various treatments, including (**a**) PANC-1 tumor-bearing mice, (**b**) 4T1 tumor-bearing mice, and (**c**) CT26 tumor-bearing mice

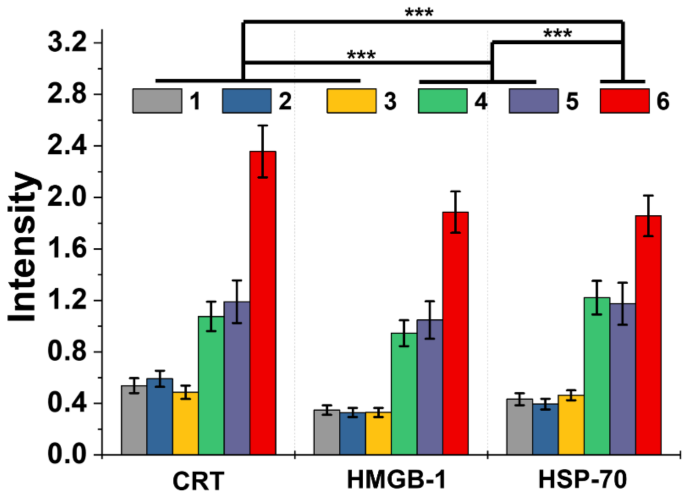

Fig. S16 Quantifications of the relative fluorescence intensity of CRT, HSP70, and HMGB1 in CT26 tumor's sections

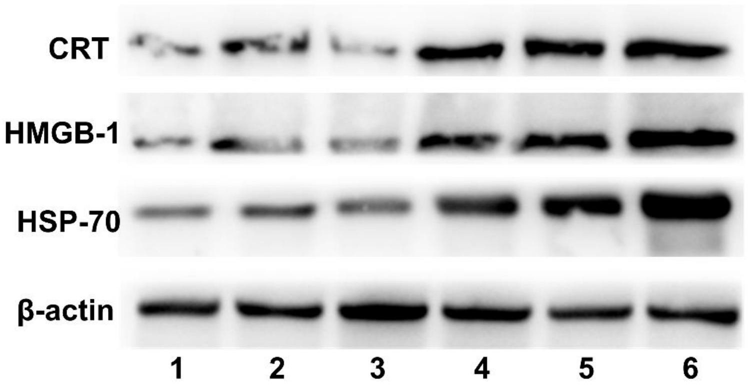

Fig. S17 The activation of DAMPs release in CT26 tumors of mice after different treatments, observed by western blot. (Saline (groups 1), laser (groups 2), ΔSt (groups 3), Cu_2O + laser plus surgery (groups 4), Cu_2O@ΔSt (group5), and Cu_2O@ΔSt + laser (groups 6))

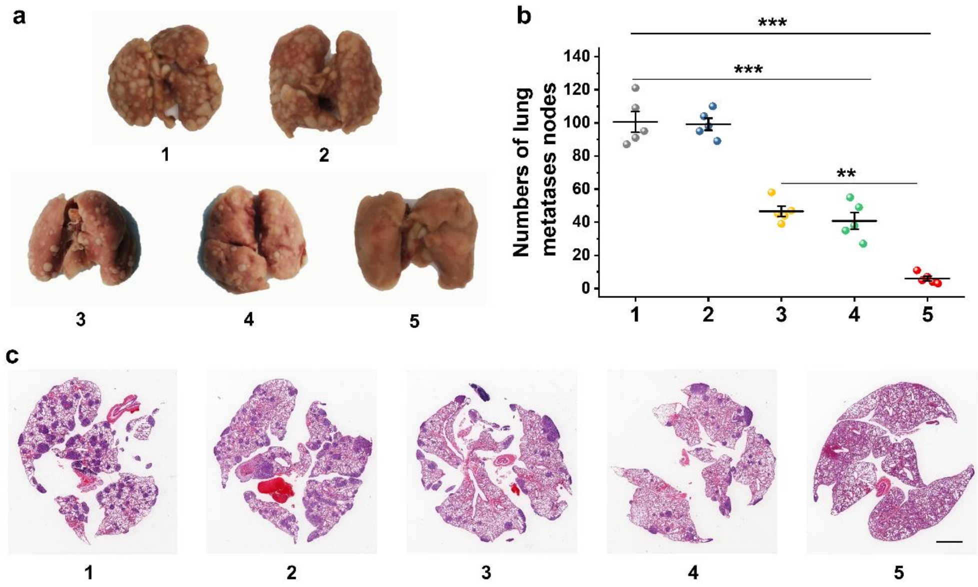

Fig. S18 (**a**) Representative photographs, (**b**) corresponding quantifications of the number of tumor nodules, and (**c**) H&E staining of lung tissue from mice after ICB treatment, in which the primary tumor of mice was removed by different methods (surgery (groups 1), bacteria plus surgery (groups 2), Cu_2O@ΔSt plus surgery (groups 3), Cu_2O + laser plus surgery (groups 4), and Cu_2O@ΔSt + laser (groups 5))

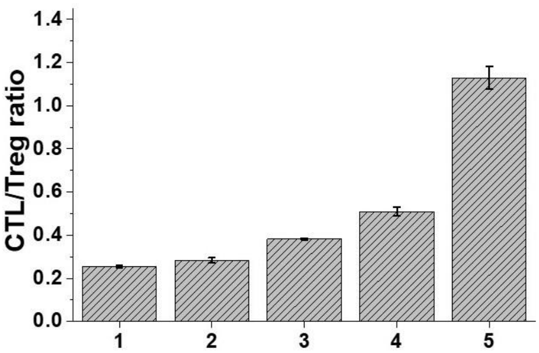

Fig. S19 Quantification of the relative CTL/Treg ratios in distant tumors of mice with primary tumor being removed by different methods (surgery (groups 1), bacteria plus surgery (groups 2), Cu_2O@ΔSt plus surgery (groups 3), Cu_2O + laser plus surgery (groups 4), and Cu_2O@ΔSt + laser (groups 5))

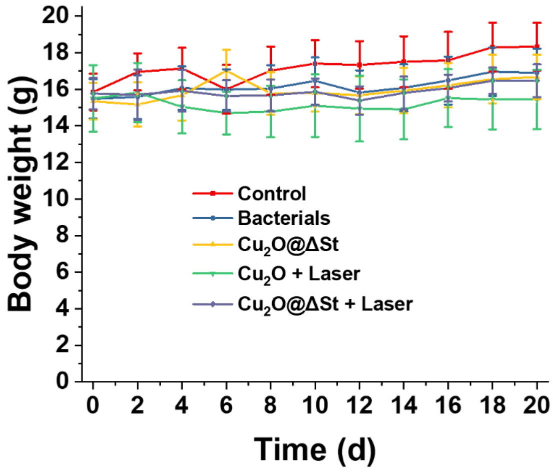

Fig. S20 Body weight variations of mice during 20 days feeding after ICB therapy

Supplementary Reference

[S1]Q. Li, P. Xu, B. Zhang, H. Tsai, S. Zheng et al., Structure-dependent electrocatalytic properties of Cu_2O nanocrystals for oxygen reduction reaction. J. Phys. Chem. C **117**(27), 13872-13878 (2013). https://doi.org/10.1021/jp403655y
